# Platelet‐Activating Factor Promotes Neutrophil Activation and Platelet–Neutrophil Complex Formation

**DOI:** 10.1111/sji.70044

**Published:** 2025-07-28

**Authors:** Lisa Wohlgemuth, Christiane Leonie Knapp, Laura Vidoni, Stefan Hug, Paul Müller, Adam Omar Khalaf Mohamed, Annika Dietz, Alexander Elias Paul Stratmann, Laura Stukan, Larissa Melina Höpfer, Bertram Dietrich Thomaß, Alexander Sebastian Koller, Frederik Münnich, Michael Ruhland, Markus Huber‐Lang, David Alexander Christian Messerer

**Affiliations:** ^1^ Institute of Clinical and Experimental Trauma Immunology University Hospital of Ulm Ulm Germany; ^2^ Department of Transfusion Medicine and Hemostaseology Friedrich‐Alexander University Erlangen‐Nuernberg, University Hospital Erlangen Erlangen Germany; ^3^ Institute of Transfusion Medicine University Hospital of Ulm Ulm Germany

**Keywords:** animal‐free ex vivo whole blood model, neutrophils, platelet‐activating factor, platelet–neutrophil complexes, thrombocytes, thromboinflammation

## Abstract

Controlling excessive inflammation remains an unmet clinical need, for example, during sepsis or after severe injuries. Platelet‐activating factor (PAF) is central in thromboinflammatory processes. However, its role in the interaction of platelets and neutrophils requires further insights. Therefore, we elucidated PAF‐related neutrophil activation, including platelet–neutrophil complex (PNC) formation and investigated potential strategies to modulate PAF‐related inflammation. For the translation of the PAF‐mediated inflammation, we applied an animal‐free human ex vivo whole blood model. The neutrophil phenotype, its function, and PNC formation were studied by flow cytometry and platelet‐related activity was assessed by light microscopy and aggregometry. PAF induced a rapid and dose‐dependent change in neutrophil phenotype, as evidenced by CD10, CD11b, and CD66b upregulation and CD62L downregulation. Moreover, PAF increased the generation of reactive oxygen species (ROS), phagocytic activity and PNC formation. Interestingly, PNCs displayed significantly enhanced ROS formation and phagocytosis compared to neutrophils without attached platelets, whereas these differences were not observed regarding phenotype changes. Furthermore, the findings were confirmed in a clinically relevant ex vivo whole blood model of lipopolysaccharide‐ or PAF‐driven inflammation. In summary, the present study elucidates PAF‐driven effects on neutrophils and their interaction with platelets. The findings might help in developing therapeutic approaches to modulate PAF‐related thromboinflammation, for example, during sepsis.

## Graphical abstract

The present study analysed the effect of the proinflammatory mediator platelet‐activating factor (PAF) on neutrophils both in vitro and in an animal‐free ex vivo whole blood model, with a particular focus on the formation of platelet–neutrophil complexes (PNCs). PAF triggered neutrophil activation (arrows), which was partially aggravated (+) or unchanged (−) by the formation of PNCs. Furthermore, potential pharmacological agents (e.g., iloprost, anti‐CD62P) and their effects on PAF‐induced platelet–neutrophil interactions and on neutrophil phenotype and effector functions were studied.

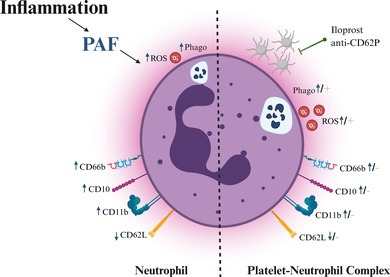

## 1 Introduction

Platelet‐activating factor (PAF) is a prominent proinflammatory and prothrombotic mediator linking inflammation and thrombosis [1, 2]. On a molecular level, PAF encompasses a variety of phospholipid mediators active at nanomolar concentrations [3, 4]. Hereafter, the present work refers to the classical structure of PAF first described by Demopoulos et al. [1, 5]. The sources of PAF are diverse because it can be produced by several cell types, including platelets, macrophages, mast cells, endothelial cells, monocytes, and granulocytes [1, 6, 7]. During inflammation, PAF is mainly synthesised via the remodelling pathway, in which PAF is generated through the acetylation of Lyso‐PAF. On the one hand, Lyso‐PAF is generated by degradation of PAF itself and/or by further hydrolyzation [2, 8]; on the other hand, Lyso‐PAF can be produced from cell membrane phospholipids through the action of phospholipase A_2_ [1].

PAF exerts effects in various tissues and on different cells. It is tempting to speculate that PAF levels differ depending on the microenvironment, because PAF plasma concentrations are increased by cytokines such as tumour necrosis factor or by activated endothelial cells [1]. In particular, within the paracellular region of platelet–neutrophil complexes (PNCs), a higher PAF concentration is likely because both cell types are able to synthesise PAF [1, 9]. Initially, PAF was described as a key platelet activator, but it also promotes the activity of leukocytes, such as neutrophil granulocytes [1, 2, 10]. PAF‐driven leukocyte activation plays a key role in promoting systemic inflammation, for example, in the context of sepsis [11], bronchial asthma [12] and coronary artery disease [13]. Neutrophil granulocytes represent a major component of innate immunity as the most abundant cell type and have a high turnover. Moreover, neutrophils serve as the first line of cellular defence against invading pathogens [14, 15, 16, 17]. To reach the extravascular site of infection or cellular damage, neutrophils can sense molecular danger and migrate via different signalling molecules as well as various adhesion receptors toward the critical zone [17, 18]. Upon arrival, neutrophils can exert different functions, including phagocytosis, the generation of reactive oxygen species (ROS; e.g., superoxide anions), the production of proteases, and the formation of so‐called neutrophil extracellular traps (NETs) [14, 15, 17, 18]. All these multifaceted functions finally enable the clearance of invading pathogens and/or cellular debris removal. Furthermore, neutrophils can modulate the immune response via cell–cell interaction with, for example, T‐cells or platelets [19].

Regarding PAF, neutrophils can generate and release PAF and react to it through their surface‐bound G‐protein‐coupled PAF receptor [20, 21]. In this regard, we and others have investigated PAF‐driven neutrophil interactions, including corresponding physiological changes and platelet–neutrophil interactions [10, 15]. PAF can induce a plethora of additional actions in neutrophils: it can increase neutrophil chemotactic activity [10, 22] and enhance their cellular polarity, motility, and adhesion capacity [23], alter membrane electrical potential [10, 24], upregulate surface‐bound integrins (CD11b), downregulate surface‐bound L‐selectin (CD62L) [10, 25, 26] and increase ROS production, intracellular pH [10, 27] and phagocytic activity [28].

Platelet and/or neutrophil activation can trigger cell–cell interaction on a humoral or cellular level. The latter includes, for example, PNC formation. Such complex formation can be triggered by different proinflammatory mediators such as PAF or lipopolysaccharide (LPS) [10, 29] and is upregulated in systemic inflammatory conditions, including COVID‐19, peritonitis, and sepsis [30, 31, 32]. PNC formation is dependent on different interactions between neutrophils and platelets, including the interaction between P‐selectin glycoprotein ligand‐1 (PSGL‐1) and P‐selectin, CD40 and CD40L, and triggering receptor expressed on myeloid cells 1 (TREM1) and TREM1L [33]. In comparison with neutrophils that are not accompanied by a platelet in direct proximity, PNCs demonstrate higher activity in terms of ROS generation and NET formation [33, 34]. Therefore, studying platelet–neutrophil interaction and the consequences thereof may help to better understand excessive thromboinflammation.

Given the clinical relevance of modulating excessive neutrophil activity, including its potential role as a coagulatory microplatform, for example, during sepsis or after severe injuries, research is needed to better understand PAF‐mediated neutrophil activation and/or PAF‐induced platelet–neutrophil interaction. This might help to further investigate potential pharmacological interventions as promising therapeutic rationales. Therefore, the present study investigated in detail the effects of PAF on neutrophils regarding changes in phenotype, cell function, and interaction with platelets. First, we described the effects of PAF exposure on neutrophils in a time‐ and concentration‐dependent manner in vitro, including the consequences on cellular activity triggered by the interaction of neutrophils with platelets. Second, we investigated the effect of the pharmacological modulation of PAF‐induced platelet–neutrophil interactions on neutrophil phenotype and effector functions. Third, in a translational approach, we validated the impact of PAF in a clinically relevant animal‐free ex vivo human whole blood model.

## 2 Methods

**FIGURE 1:**
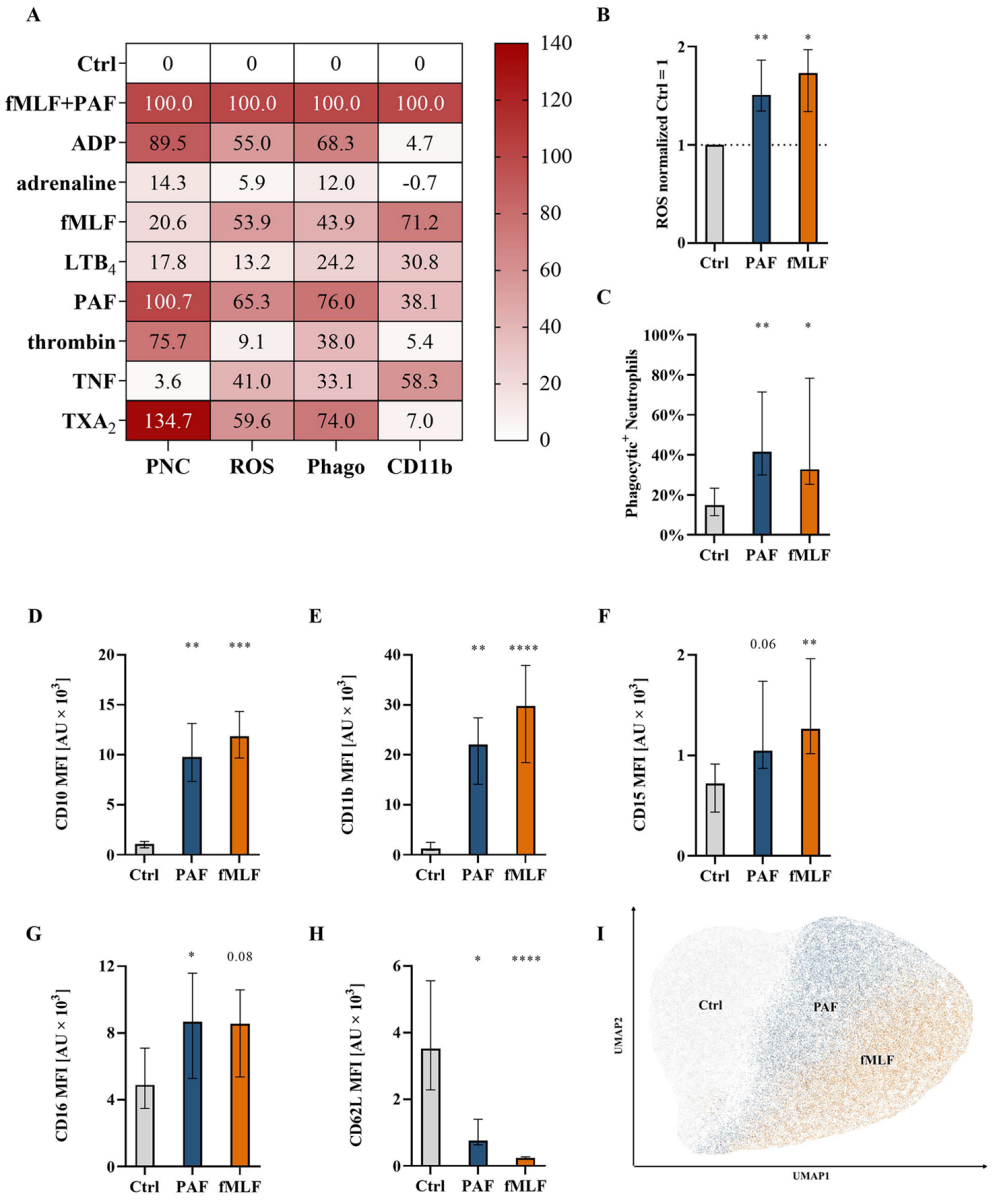
PAF‐induced effects on neutrophil activation and changes in neutrophil phenotype in diluted whole blood in vitro. (A) Heatmap of the impact of various mediators on neutrophil phagocytic activity, reactive oxygen species (ROS) generation, platelet–neutrophil complex (PNC) formation and CD11b expression within ‘conf1’. The value 0% corresponds to neutrophils stimulated with PBS^++^ as control (Ctrl) and 100% represents the maximum effect of neutrophils stimulated with an inflammatory cocktail containing PAF and fMLF. *n* = 5–7, median values. Within (A), samples were stimulated with the following concentrations: Adenosine diphosphate (ADP) 5 μM, adrenaline 1 ng/mL, fMLF 1 μM, leukotriene B_4_ (LTB_4_) 0.1 μM, PAF 1 μM, thrombin 5 U/mL, tumour necrosis factor (TNF) 1.1 μM, or thromboxane A_2_ (TXA_2_) 2 μM, or co‐stimulated with PAF and fMLF and measured by flow cytometry. (B) ROS generation, (C) phagocytic activity, (D) CD10, (E) CD11b, (F) CD15, (G) CD16, (H) CD62L, and (I) Uniform Manifold Approximation and Projection (UMAP) analysis of Ctrl, PAF and fMLF (from data of D–H). (B) illustrates the increase in ROS production normalised to unstimulated neutrophils (Ctrl = 1.0). (C) shows the percent positive neutrophils for phagocytosis. (D–H) The *y*‐axis reports median fluorescence intensity (MFI) for all CD molecules. In (B) the following concentrations were used: PAF 1 μM, fMLF 10 μM. (B–I) *n* = 7–9 median ± interquartile range. *p < 0.05, **p < 0.01, ***p < 0.001, and ****p < 0.0001, respectively. Friedmann‐test was applied for values before normalisation in (B), Kruskal‐Wallis test for (C–H) using neutrophils stimulated with PBS^++^ as control (Ctrl) versus stimulated with PAF or fMLF, all with uncorrected Dunn's test. Further analysis is illustrated in Figure S2.

**FIGURE 2:**
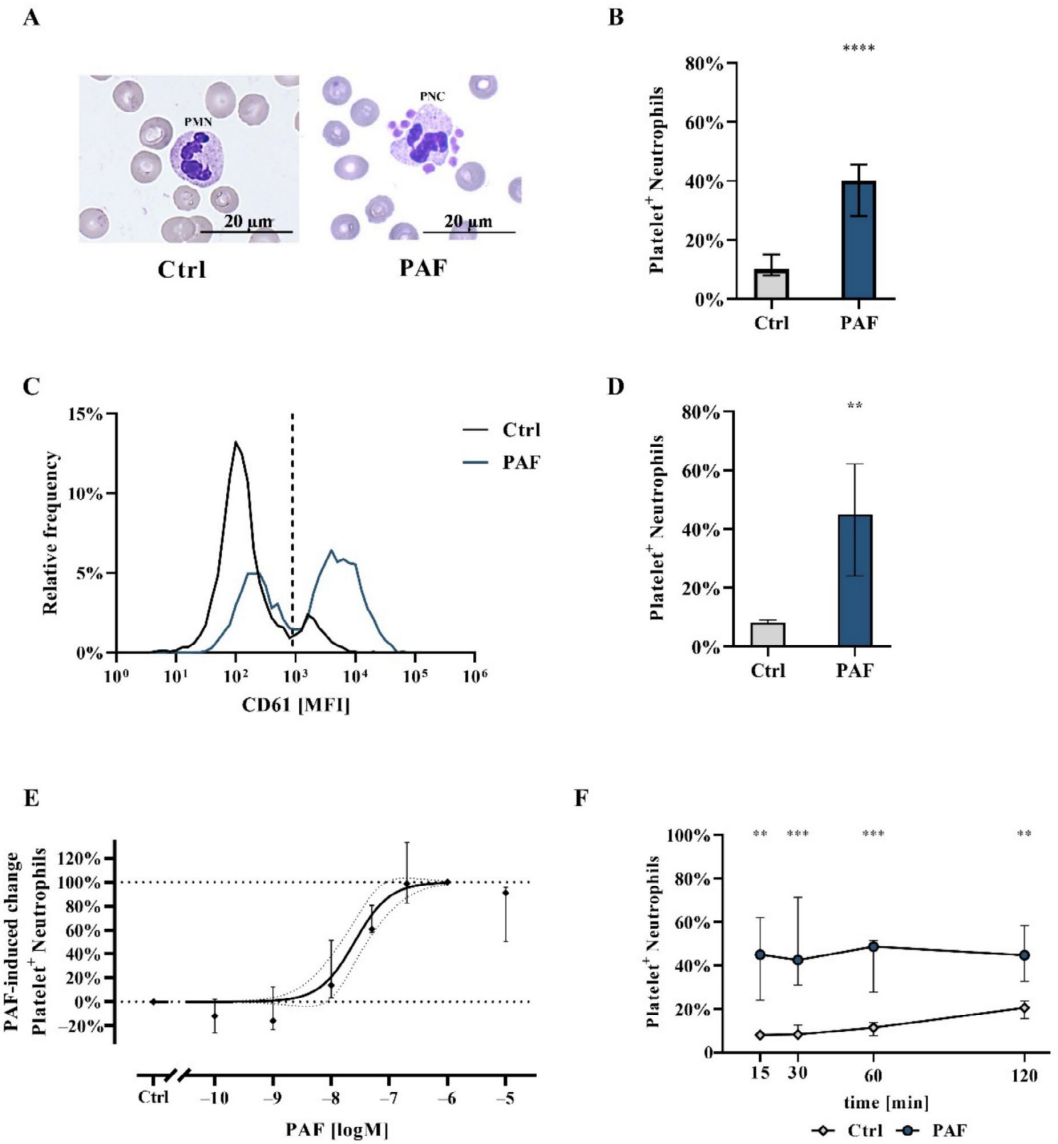
In‐depth analysis of platelet–neutrophil complex (PNC) formation after stimulation with PAF in diluted whole blood in vitro. (A) Representative light microscopy of neutrophil not in complex with platelets (PMN, PBS^++^‐stimulated Ctrl) and an exemplary PNC (PAF‐stimulated) and (B) summary data for the percentage of PNCs detected by light microscopy and manual counting. (C) Representative flow cytometry gating using CD61 median fluorescence intensity (MFI) for PNCs after stimulation with PBS^++^ as control (Ctrl) or PAF and (D) summary data for the percentage of PNCs detected by flow cytometry. (E) Concentration and (F) time dependency of PNC formation after PAF stimulation. For the concentration dependency, neutrophils were stimulated with PAF concentrations from 10 μM to 100 pM. The value 0% corresponds to neutrophils stimulated with PBS^++^ as control (Ctrl) and 100% represents the maximum effect of neutrophils stimulated with PAF. For all other analyses the used PAF concentration was 1 μM. *n* = 5–10, median ± interquartile range. **p < 0.01, ***p < 0.001, and ****p < 0.0001, respectively. Mann–Whitney *U*‐test for (B, D, F) neutrophils stimulated with PBS^++^ as control (Ctrl) versus neutrophils stimulated with PAF and in (F) for each time point (15, 30, 60 and 120 min).

**FIGURE 3:**
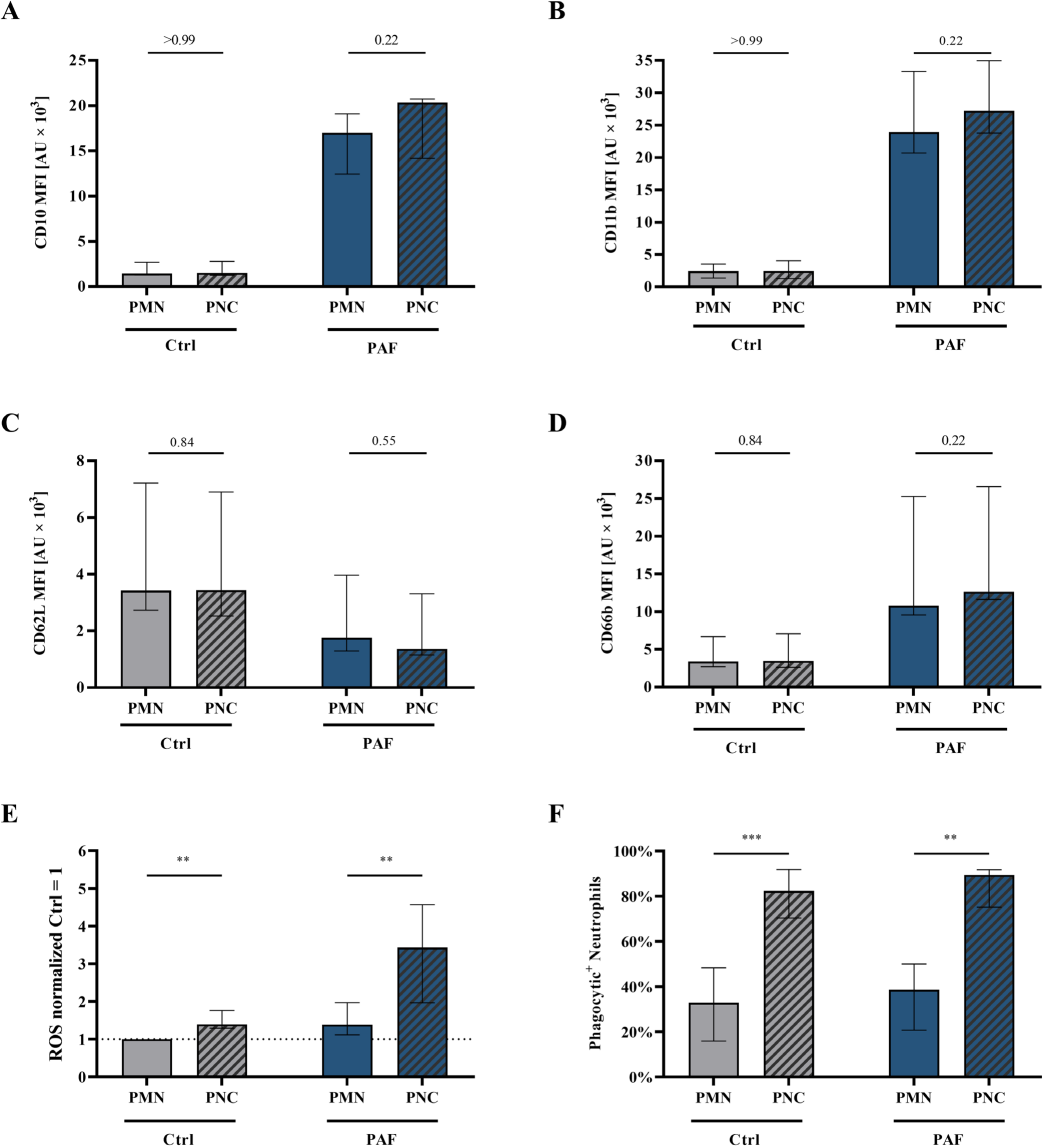
Platelet–neutrophil‐complex (PNC) formation affects neutrophil function but not its phenotype in diluted whole blood in vitro after PAF stimulation compared to neutrophils not in a complex with platelets (PMN). (A) CD10, (B) CD11b, (C) CD62L, (D) CD66b, (E) generation of reactive oxygen species (ROS), and (F) phagocytic activity. (A–D) The *y*‐axis reports median fluorescence intensity (MFI) for all CD molecules, (E) shows the increase in ROS production normalised to unstimulated PMN (Ctrl = 1) and (F) shows the percent positive neutrophils for phagocytosis. The used PAF concentration was 1 μM. *n* = 7–9, median ± interquartile range. **p < 0.01 and ***p < 0.001, respectively. Mann–Whitney *U*‐test for (A–D, F), Wilcoxon‐test applied to values before normalisation for (E), with PMN stimulated with PBS^++^ as control (Ctrl) versus PNC stimulated with PBS^++^ (Ctrl) and PMN stimulated with PAF versus PNC stimulated with PAF. Further analysis is illustrated in Figure S6.

**FIGURE 4:**
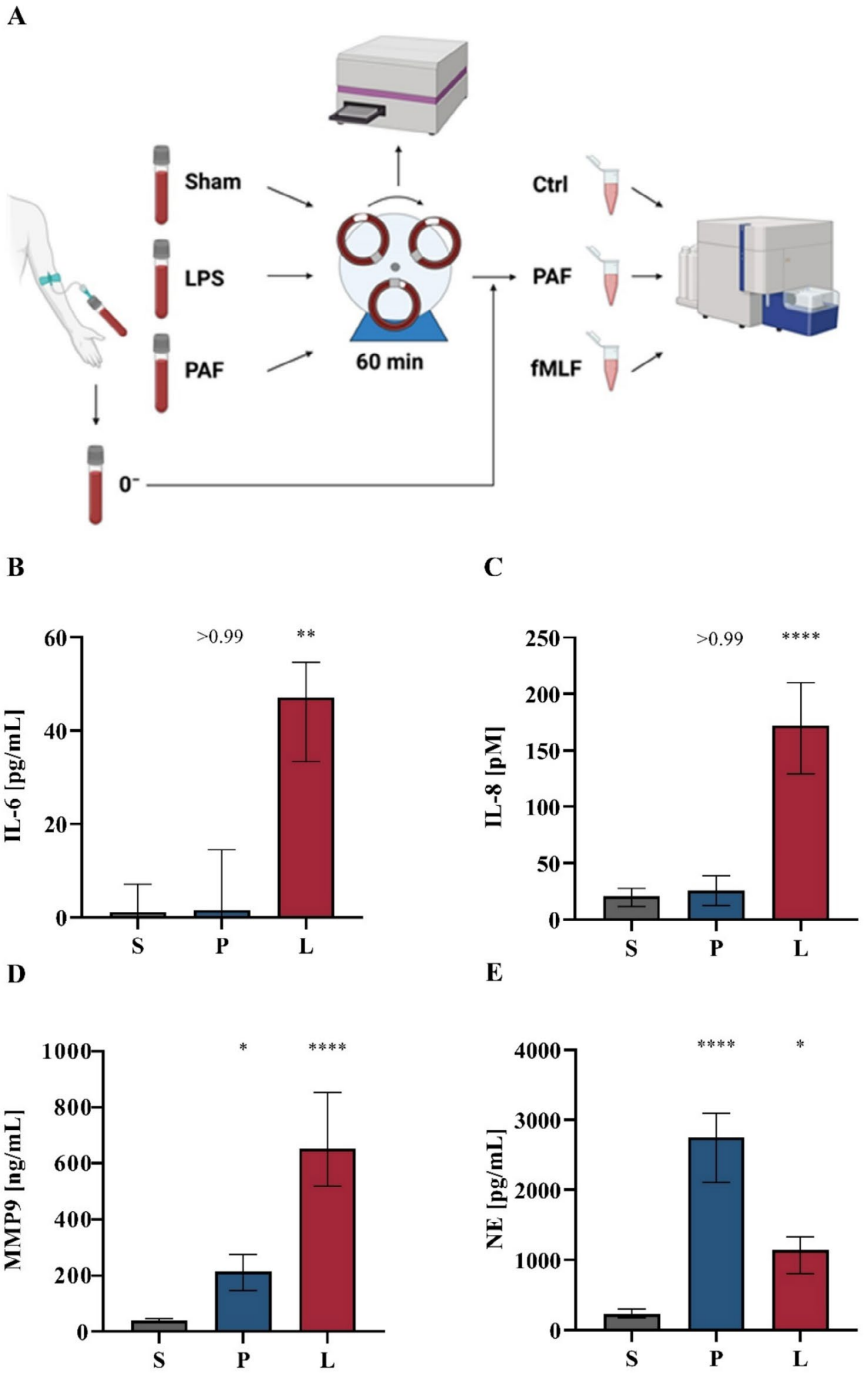
PAF‐driven inflammation does not affect humoral inflammation within an animal‐free ex vivo whole blood model. (A) Human whole blood was either incubated within the whole blood model for 1 h either with PBS^++^ as Ctrl (Sham (S)), PAF (P), or LPS (L), or directly analysed without exposure to the whole blood model (0^−^). Plasma samples were analysed regarding the levels of (B) interleukin‐6 (IL‐6), (C) interleukin‐8 (IL‐8), (D) matrix metallopeptidase 9 (MMP9), and (E) neutrophil elastase (NE). The following concentrations were used: PAF 1 μM, LPS 100 ng/mL. *n* = 10, median ± interquartile range. *p < 0.05, **p < 0.01, and ****p < 0.0001, respectively. Kruskal‐Wallis‐test with uncorrected Dunn's test for neutrophils incubated with PBS^++^ as control (Ctrl) versus neutrophils incubated with PAF or LPS.

**FIGURE 5:**
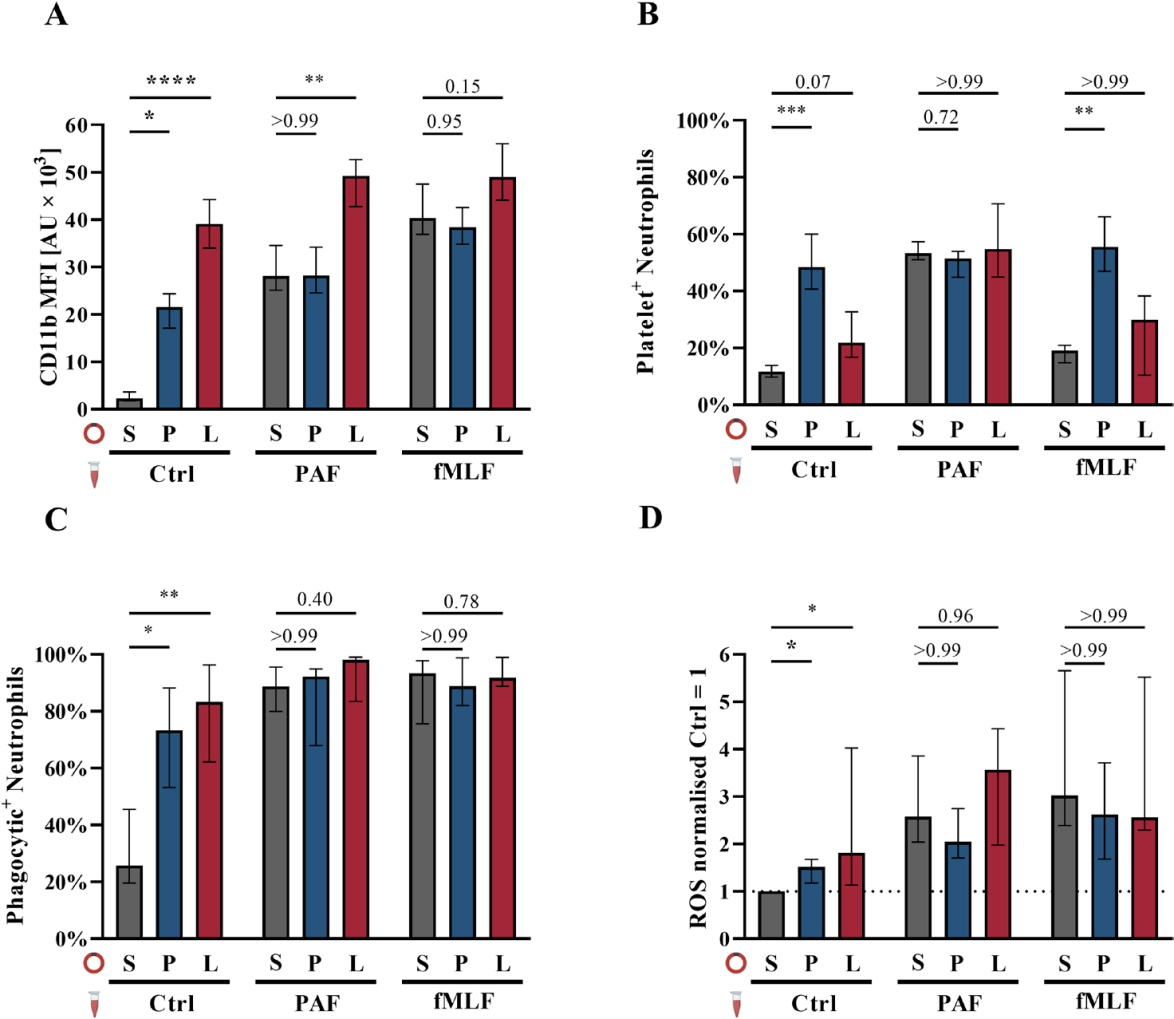
PAF‐driven inflammation changes neutrophil function within an animal‐free ex vivo whole blood model. Human whole blood was either incubated within the whole blood model for 1 h with PBS^++^ as Ctrl (Sham (S)), PAF (P), or LPS (L), or directly analysed without exposure to the ex vivo whole blood model (0^−^) as indicated in Figure 4A. Neutrophils in diluted whole blood were further stimulated with PBS^++^ as Ctrl, PAF, or fMLF in vitro for 15 min. (A) CD11b expression, (B) platelet–neutrophil complex (PNC) formation, (C) phagocytic activity, and (D) generation of reactive oxygen species (ROS). The following concentrations were used: PAF 1 μM, fMLF 10 μM, and LPS 100 ng/mL. *n* = 10, median ± interquartile range. *p < 0.05, **p < 0.01, ***p < 0.001 and ****p < 0.0001, respectively. Percent positive neutrophils for PNC formation in (B) and phagocytic activity in (C). (D) Shows the increase in ROS production normalised to unstimulated PMN (Ctrl = 1.0). Kruskal‐Wallis test (A–C) and Friedmann‐test for absolute values (D) for neutrophils incubated with PBS as control (Ctrl) versus neutrophils incubated with PAF or LPS, all with uncorrected Dunn's test. Further analysis is illustrated in Figure S12.

This paper contains three data sets: the first, termed the reference data set (hereafter referred to as ‘ref’), includes all experiments conducted to monitor the PAF‐induced response of neutrophils and platelets (Figures 1B–I, 2, 3, 4, 5, Figures S3, S7–S12). The second data set (hereafter referred to as ‘conf1’) as well as the third data set (hereafter referred to as ‘conf2’) consist of confirmatory data derived from other large experimental approaches (‘conf1’: Figure 1A, Figure S6; ‘conf2’: Figure S2). The two confirmatory data sets were utilised to conserve resources, to reduce the burden to blood donors, and for economic reasons. They mostly present findings using different antibodies, devices, and protocols.

### 2.1 Blood Sampling

Blood was donated by healthy human volunteers of both sexes between the ages of 18 and 35 years (‘ref’) and 20 and 59 years (‘conf1’ and ‘conf2’). All experiments were performed in conformity with the Helsinki declaration [35]. Following ethical approval (‘ref’: #459/18, Local Independent Ethics Committee of the University of Ulm; ‘conf1’ and ‘conf2’: #343_18 B, Local Independent Ethics Committee of the University Hospital of Erlangen) and obtaining written informed consent, blood was drawn by peripheral venipuncture in accordance with the guidelines of the World Health Organisation [36] and collected into monovettes containing 3.2% trisodium citrate (Sarstedt, Nümbrecht, Germany) or 16 IE/mL lithium‐heparin (Sarstedt). In general, blood stasis was limited to a maximum of 60 s prior to puncture, and at least the first 2–3 mL were immediately discarded. Following blood collection, the samples were maintained at room temperature and used for all experiments within 20 min.

### 2.2 Measurement of PAF‐Induced Changes in Neutrophil Function and Phenotype

For stimulation and subsequent staining, anticoagulated blood (‘ref’ neutrophil phenotype 10 μL citrate‐anticoagulated blood, ‘ref’ neutrophil function: 10 μL heparin‐anticoagulated blood; ‘conf1’: 10 μL heparin‐anticoagulated blood, ‘conf 2’: 50 μL heparin‐anticoagulated blood) was added to phosphate‐buffered saline containing calcium and magnesium (PBS^++^, #14040‐091, Thermo Fisher Scientific, Darmstadt, Germany) adjusted to pH 7.3. The total combined volume of blood, stimuli, and staining reagents was ‘ref’: 50 μL, ‘conf1’: 50 μL, and ‘conf2’: 100 μL.

Blood samples were subsequently stimulated with either PAF (PAF C‐16, 1 μM if not indicated otherwise, #18779, Cayman Chemical, Ann Arbor, MI, USA), N‐formylmethionyl‐leucyl‐phenylalanine (fMLF, 10 μM if not indicated otherwise, #F3506, Sigma Aldrich, Steinheim, Germany), adenosine 5′‐diphosphate (ADP, 5 μM, #A2754, Sigma Aldrich), adrenaline (1 ng/mL, 1:1000, InfectoPharm Arzneimittel und Consilium GmbH, Heppenheim, Germany), leukotriene B_4_ (LTB_4_, 0.1 μM, #20110, Cayman Chemical), recombinant tumour necrosis factor (TNF, 1.1 μM, #570102, Biolegend, San Diego, CA, USA), thrombin (5 U/mL, # 13188, Cayman Chemical), thromboxane A_2_ (TXA_2_, 2 μM, U‐46619, #19020, Cayman Chemical), or PBS^++^ as control. Moreover, blood samples were stimulated with an inflammatory cocktail containing 1 μM PAF and 0.01 μM fMLF with the final concentrations as indicated above.

Prior to stimulation, the blood and the tubes used were acclimatised at 37°C in a water bath. The samples containing blood, buffer, and stimuli beads were incubated for ‘ref’ in a light‐protected water bath at 37°C. Subsequently, samples were stained with respective fluorescent‐labelled antibodies or reagents for 15 min for ‘ref’ phenotype and 30 min for ‘ref’ function under the same incubation conditions as before. For ‘conf1’ and ‘conf2’, the samples contained not only blood, buffer and stimuli, but also the antibodies directly rather than subsequently added and were incubated for 30 min in a light‐protected water bath at 37°C. Taken together, the blood samples and corresponding tubes were kept as closely as possible to 37°C to mimic standard body temperature.

The antibodies (and their respective isotypes) as well as other fluorescent probes and their corresponding final concentrations are listed in Table S2. After incubation, each sample was individually transferred to polystyrene round‐bottom tubes (#352052, Corning Science México S.A. de C.V., Reynosa, Mexico) containing 1 mL of a 1:10 dilution with distilled water of FACS Lysing Solution (#349202, BD Biosciences), and chemically fixed for 30 min in the dark. Subsequently, the samples were centrifuged at 340×*g* for 5 min, the supernatant was discarded, and the pellet was resuspended in 100 μL PBS without calcium or magnesium (PBS^−−^, Thermo Fisher Scientific) containing 1% bovine serum albumin (#A8022, Sigma‐Aldrich). Finally, the samples were maintained at 4°C in the dark until measurement within 1 h.

### 2.3 Determination of PAF Concentration‐Dependency In Vitro

For the PAF‐induced concentration‐dependency experiments, the same coagulant and volume as described for the ‘ref’ neutrophil phenotype and function was used. Neutrophils were stimulated with PAF at varying concentrations as follows: 10 μM, 1 μM, 200 nM, 50 nM, 10 nM, 1 nM, and 100 pM. Furthermore, the used antibody/reagent concentrations were congruent as listed for the ‘ref’ data set in Section 2.2.

### 2.4 Measurement of PAF Time‐Dependency In Vitro

To determine the effects of PAF in a time‐dependent manner, whole blood was stimulated with PAF for 15, 30, 60, and 120 min. Each condition included the fluorescent‐labelled antibodies or reagents (CellROX for ROS generation, phagocytosis beads for phagocytic activity) for the last 15 min of the incubation time as listed for the ‘ref’ data set in Section 2.2.

### 2.5 Pharmacological Interventions of PAF‐Induced Effects In Vitro

When analysing the effects of modulators on whole blood, 10 μL citrate‐anticoagulated blood was used for monitoring neutrophil phenotype changes, and 10 μL lithium‐heparin‐anticoagulated blood was used to capture functional changes. The blood was also incubated with the respective pharmacological agents or PBS^++^ as control. Pharmacological inhibitors and antagonists were selected as a broad and brief exploratory approach to modulate neutrophil and/or platelet activity in the context of acute PAF‐induced inflammation. The following final concentrations of the pharmacological agents were used: acetylsalicylic acid (ASA, targeting cyclooxygenase 1 on platelets [37], 1.7 μM, #A5376, Sigma‐Aldrich), GW4869 (blocking exosome generation by platelets [38], 2 μM, #D1692, Sigma‐Aldrich), iloprost (targeting the prostaglandin I_2_ receptor on platelets [39], 1 μM if not indicated otherwise, #SML1651, Sigma‐Aldrich), N‐acetyl‐L‐cysteine (inhibits platelet aggregation [40], 2 mM, #A7250, Sigma‐Aldrich), suramin (antagonist of ADP‐induced platelet aggregation [41], 100 μM, # S2671, Merck, Darmstadt, Germany), zileuton (5‐lipoxygenase/leukotriene inhibitor [42], 2.5 μM, #A64077, Cayman Chemical), or anti‐CD62P (targeting P‐selectin on platelets, 60 nM if not indicated otherwise, #304904, BioLegend). The samples were incubated for 10 min at 37°C in a light‐protected water bath prior to stimulation with 1 μM PAF. Likewise, to investigate the effect on a priori stimulation, blood was exposed to either LTB_4_ (100 nM, #71160‐24‐2, Cayman Chemical), serotonin (500 ng/mL, #14927, Cayman Chemical), or TXA_2_ (2 μM, #D8174, Merck) before PAF stimulation. After PAF stimulation, samples were stained with fluorescent‐labelled antibodies or reagents and further processed according to Section 2.2.

### 2.6 Animal‐free Ex Vivo Whole Blood Model

The concept of the human whole blood model has previously been described [29]. In the present work, whole blood was sampled in standard neutral monovettes (Sarstedt) supplemented with 0.5 I.E. heparin per mL. Either PBS^++^ (Sham, ‘S’), PAF (1 μM), or LPS (100 ng/mL, from *Escherichia coli* O55:B5, #L2637, Sigma Aldrich) was added to the monovettes. An additional sample was directly processed for flow cytometry and had no contact with the model (depicted as ‘0^−^’).

After sampling, 9 mL of the blood was transferred to 33 cm long heparin‐coated tubes (Cortiva, #M999413C, Medtronic, Meerbusch, Germany). The ends of the tube were connected to a circuit using a similarly coated connector (Cortiva, #CB4629, Medtronic). An air bubble of approximately 1.5 mL was left inside the system to generate a continuous circulation of the blood. The loops were attached to a spinning wheel (Snijders Labs, Tilburg, Netherlands) rotating at 7 rpm. The system was incubated for 60 min in an incubator at 37°C. Following the incubation period, the tubing loops were cut open. Initially, 95 μL blood was directly drawn into dry‐heparin‐anticoagulated glass capillary tubes for blood gas analysis (ABL 800 Flex, Radiometer GmbH, Krefeld, Germany). Subsequently, 1 mL of the remaining blood was transferred into a heparin‐anticoagulated tube (Sarstedt) for the analysis of phagocytotic activity and ROS generation. The remaining blood was transferred into citrate anticoagulated monovettes (Sarstedt) for the analysis of all other parameters. Sodium, potassium, ionised calcium, lactate, glucose, and blood pH were determined using a standard blood gas analyser (ABL 800 Flex, Radiometer GmbH). The differential blood count was determined using a standard haematology analyser (Sysmex CN 2000, Sysmex, Kobe, Japan) according to the manufacturer's standard protocol. To obtain plasma, the citrate anticoagulated blood was centrifuged for 10 min at 400×*g* at room temperature and stored at −80°C until further use. The determination of PAF‐induced changes in neutrophil phenotype and function was performed as described in Section 2.2. The blood samples of the whole blood model after the 1‐h incubation on the spinning wheel were either stimulated with PBS^++^ as buffer control (Ctrl), PAF (1 μM), or fMLF (10 μM).

### 2.7 Determination of PNCs by Light Microscopy and Flow Cytometry

PNCs were analysed by light microscopy and flow cytometry as previously described [10, 29, 43]. For the analysis by light microscopy (Axio Imager M1, Carl Zeiss, Oberkochen, Germany), 250 μL citrate‐anticoagulated whole blood was either diluted with 250 μL PBS^++^ adjusted to pH 7.3 for Ctrl or with the same volume containing a final concentration of 1 μM PAF. The samples were incubated for 15 min at 37°C on a spinning wheel at approximately 7 rpm. Subsequently, blood smears were made and stained with the ‘Hemacolor Rapid staining of blood smear—staining set for microscopy’ (#111661, Sigma Aldrich). A minimum of 50 neutrophils per specimen were analysed by two independent and blinded individuals. Each neutrophil with at least one platelet in direct proximity (< 2 μm) was counted as a PNC. A representative PNC identified by light microscopy is provided in Figure 2A. The flow cytometric analysis was conducted by the general gating strategy to identify neutrophils (according to Section 2.9), and PNCs were identified as described in Section 2.2. An example of the gating strategy is shown in Figure 2C.

### 2.8 Effects on Platelet Activation Within the Ex Vivo Whole Blood Model

To assess the effects on platelet activation within the ex vivo whole blood model, 100 μL citrate‐anticoagulated whole blood of either the Ctrl (Sham, ‘S’), PAF, or LPS loop was diluted with 1125 μL Hanks' Balanced Salt Solution (HBSS^++^, #14025‐050, Thermo Fisher Scientific) adjusted to pH 7.3. Subsequently, 10 μL of the diluted whole blood with a total combined volume of blood, stimuli, and staining reagents of 50 μL was each stimulated with PBS^++^ as Ctrl or PAF (1 μM) for 5 min at 37°C in a light‐protected water bath. Subsequently, blood samples were stained with fluorescent‐labelled antibodies for 5 min under the same incubation conditions. The following antibodies (with respective isotypes), both from BioLegend, with corresponding final concentrations were used: anti‐CD62P (P‐selectin, FITC, 8 μg/mL, #304904; isotype #400108) and anti‐CD61 (GPIIIa, PerCP, 2 μg/mL, #336410; isotype #400148). Following incubation, the samples were transferred to polystyrene round‐bottom tubes containing 950 μL HBSS^++^ and immediately measured on the flow cytometer according to the procedure described in Section 2.9.

### 2.9 Flow Cytometry

Doublets were removed by analysing the linearity of FSC‐A versus height (FSC‐H). Granulocytes were mainly identified based on their forward (FSC‐A) and side scatter area (SSC‐A) properties. For the blood samples used in the ex vivo whole blood model, a complete blood count confirmed that granulocytes consisted mainly (usually > 95%) of neutrophil granulocytes. Moreover, all analysed parameters were unimodally distributed (expect phagocytic activity and PNC formation, for technical reason). Therefore, and because median values were analysed, and the same blood taken at the same time from the same blood donors was used, granulocytes were analysed and henceforth are referred to as neutrophils. The gating strategy and representative histograms are illustrated in Figure S1. For all antigens, appropriate isotype controls and single‐staining controls were performed (data not shown). Furthermore, for all experiments, a minimum of 3000 neutrophils were recorded using a BD FACSLyric flow cytometer (BD Biosciences) with the BD FACSuite application software (all cohorts). To determine the changes in platelet activation within the ex vivo whole blood model (Section 2.8), the measured samples were gated by FSC‐A and SSC‐A using a logarithmic scale. Subsequently, only CD61‐positive cells were used for analysing the antigen expression of CD62P (representative gating provided in Figure S11A).

### 2.10 Determination of Platelet Aggregation

Platelet aggregation was determined by impedance aggregometry (Multiplate analyser Version 1.0, Roche Diagnostics GmbH, Mannheim, Germany). Hirudin‐containing whole blood was used and incubated with PBS^++^, thrombin receptor activating peptide 6 (TRAP‐6, 15 μM, # T5045‐02A.5 United States Biological, Salem, USA), PAF (1 μM), or fMLF (1 μM) during the measurement as indicated by the manufacturer. The multiplate analyser test cells (Roche Diagnostic GmbH) were covered by the aggregated platelets and increased the electrical conductivity, which was recorded by the device. The aggregation units (AGU) per minute were recorded for 6 min.

### 2.11 ELISA

Measurement of plasma levels of matrix metallopeptidase 9 (MMP9, #DY911, R&D Systems, Minneapolis, USA), interleukin‐6 (IL‐6, #555220, BD Bioscienes), IL‐8 (#DY208, R&D Systems), and neutrophil elastase (NE, #DY9167‐05, R&D Systems) was conducted using citrate‐anticoagulated plasma and by standard enzyme‐linked immunosorbent assay (ELISA) as indicated by the manufacturer.

### 2.12 Data Analysis and Statistics

The flow cytometry data were analysed using the custom‐written, python‐based flow cytometry analytics software ‘BFlow’ (BFlow Project, www.bflow.science, last accessed 1 June 2024). All data was explored in single data points, however, to limit graphic size and to improve readability, the data is presented as median with error bars indicating the interquartile range, for example, median (25^th^ percentile; 75^th^ percentile). Data analysis was performed with licensed versions of Microsoft Excel 2019 (Microsoft, Redmond, USA) and GraphPad Prism 9 (GraphPad Software Inc., San Diego, USA). In Figure 1I, neutrophil subgroups were analysed using Uniform Manifold Approximation and Projection (UMAP), implemented in Scanpy version 1.11.1, and run with Python 3.12.8. UMAP embedding was conducted for two dimensions (UMAP1 and UMAP2) using the first three principal components obtained from a preceding Principal Component Analysis (PCA). The number of neighbours for constructing the UMAP graph was set to 20, while all other parameters were kept at their default values. Figure 4 was created in part with biorender.com. To calculate the half‐maximal effective concentration (EC_50_), data was normalised to buffer control (Ctrl) = 0% (bottom) and PAF 1 μM = 100% (top). The Ctrl was included in the EC_50_ calculation as two logarithmic steps below the lowest LPS concentration. EC_50_ was calculated using the formula: $Y=\text{bottom}+\frac{\mathrm{top}-\text{bottom}}{1+10^{(\log\left({\mathrm{EC}}_{50})-X\right)\times \text{HillSlope}}}$ with *Y* = response, *X* = decadic logarithm of the concentration, and top and bottom = plateaus in the units of the *Y*‐axis. For the calculation of a summarised EC_50_ combining multiple donors, the EC_50_ values were calculated combining all the respective experiments in one fit and is reported as the best‐fit value with a 95% confidence interval (95% CI). In statistical testing, data were considered to be unpaired and non‐parametrically distributed except when analysing the ROS generation, which was considered to be paired.

## 3 Results

### 3.1 PAF Elicits a Concentration‐ and Time‐Dependent Change in Neutrophil Phenotype and Cellular Activity

As initial characterisation of the effects of PAF exposure on neutrophils, PAF and other inflammatory mediators were compared regarding PNC formation, ROS generation, phagocytic activity and CD11b expression (Figure 1A). Interestingly, some mediators such as ADP and TXA_2_ mainly increased PNC formation, ROS generation and phagocytic activity, whereas CD11b expression remained largely unaltered. While fMLF and TNF similarly increased ROS generation and phagocytic activity, by contrast they also increased CD11b expression, whereas no PNC formation was observed. PAF was a potent inductor of PNC formation. In addition, it increased ROS generation and phagocytic activity while in parallel it also (mildly in comparison to fMLF) augmented CD11b expression on neutrophils. It is noteworthy that simultaneous exposure to PAF and fMLF displayed additive effects on neutrophil stimulation. In a more detailed analysis, PAF was compared to fMLF within a wider set of parameters (Figure 1B–I). Here, PAF and fMLF elicited similar effects on phagocytic activity and ROS as well as on several activation markers. The PAF‐induced effects on neutrophils were confirmed in a further set of experiments (Figure S2). The different sets of in vitro experiments were conducted in samples obtained from healthy volunteers without any acute or chronic medication and no signs of infection, which highlights the reliable applicability within healthy physiological conditions. The effort of conducting the experiments in different locations and instruments emphasises even more the robustness and given validity of the results obtained; however, limitations such as young and healthy blood donors, as discussed below, should be considered.

In the following step, the concentration‐dependency of the PAF‐induced effects was assessed (Figure S3). In most cases, 1 μM was identified as the PAF concentration with the greatest effect. In detail, the EC_50_ with their respective 95% CI were determined for CD10 (10.3 nM, 6.9–14.9), CD11b (14.7 nM, 10.3–20.7), CD62L (79.2 nM, 63.7–98.8), CD66b (15.4 nM, 10.9–21.4), phagocytic activity (19.2 nM, 12.7–29.0) and ROS generation (55.3 nM, 38.9–69.5). Furthermore, kinetic properties of the PAF‐induced effects on neutrophils were determined. Most phenotype and functional parameters showed a rapid increase 15 min after PAF stimulation, with peaks recorded after 30 min of PAF exposure. Moreover, some (CD10, CD15, CD16) but not all markers (CD11b, CD62L, phagocytic activity) returned to the levels of unstimulated neutrophils during the study period (Figure S4).

### 3.2 PAF Triggers PNC Formation

In the initial comparison of inflammatory mediators (Figure 1), PAF‐induced PNC generation was observed, which was in accordance with previous data [10]. Figure 2 reports on the detailed characterisation of this PNC formation. Flow cytometry and light microscopy showed similar levels of PNC formation. Exposure of diluted whole blood to PAF not only increased the frequency of PNC formation, but also resulted in an increased number of platelets per PNC (Ctrl: 1 (1;1) vs. PAF 4 (3;5)). PNC formation displayed a clear concentration dependency (EC_50_ 25.3 nM (15.3–41.4)). PAF‐mediated PNC formation was demonstrated to peak at 15 min within the analysed time points, with a sustained PAF‐elicited PNC formation after 120 min. As presented above (Figure 1), PAF but not fMLF induced PNC formation, which coincided with a similar activation pattern on determining thrombocyte activation by impedance aggregometry (Figure S5) and CD62P expression on platelets measured by flow cytometry (Ctrl: 0.3% (0.2; 0.4), PAF: 18.1% (10.9–24.2), fMLF: 0.4% (0.2–0.5) and TRAP‐6: 92.6% (91.0–95.8), data not shown).

### 3.3 PAF‐Induced PNC Formation Increases Neutrophil Functional Activity but Negligibly Impacts the Neutrophil Phenotype

To further characterise the impact of PAF‐induced PNC formation, the PNC phenotype and the cellular activity were analysed compared to neutrophils without adherent platelets (hereafter referred to as PMNs). PNCs did not differ from PMNs in terms of CD10, CD11b, CD62L, or CD66b (Figure 3, CD11b confirmed in an additional data set, Figure S6). By contrast, in diluted blood with and without exposure to PAF, PNCs exhibited significantly enhanced ROS production as well as phagocytic activity (Figure 3, confirmed in an additional data set, Figure S6).

### 3.4 Effective Pharmacological Modulation of the PAF‐Induced Activity of Neutrophils

Due to the PNC‐mediated alterations in neutrophil activity, several potential pharmacological modulators of PNC formation were analysed regarding their interaction on PAF exposure (Figure S7). Herein, the screening revealed significant inhibition of PNC formation by the pharmacological agent iloprost, as well as by an antibody against CD62P. The inhibitory effects of iloprost and the blockage of CD62P displayed a clear concentration dependency (Figure S8). Although iloprost and blockage of CD62P also decreased phagocytic activity, both modulators did not affect ROS generation. In accordance with the previously mentioned PNC effects on neutrophil phenotype (Figure 3), iloprost and blocking CD62P did not modulate selected neutrophil activation markers (Figure S9). Furthermore, no additive effects were observed by parallel stimulation of neutrophils with PAF, except for an additive effect of PAF and TXA_2_ costimulation on PNC formation (Figure S10).

### 3.5 Translation in an Animal‐free Human Ex Vivo Whole Blood Model: Analysis of the PAF‐Induced Inflammation and Comparison to Endotoxemia

The PAF‐induced effects on neutrophils were further investigated in a clinically relevant ex vivo whole blood model. Here, whole blood was exposed to buffer control (PBS^++^), PAF, or LPS for 1 h to mimic acute inflammation (Figure 4). Moreover, blood from the whole blood model was further stimulated in vitro to briefly simulate acute inflammation with different combinations of inflammatory mediators.

In accordance with previous data [29], exposure of whole blood to the tubing system of the model did not alter the cell count, including leukocytes and thrombocytes, in a relevant manner (Table S1). In addition to an expected slight decrease in glucose and an increase in lactate, the pH, as well as the carbon dioxide and oxygen partial pressures, remained largely stable within common physiologic venous ranges (Table S1). Moreover, the platelet count and CD62P expression (0^−^: 6.2% (4.4; 9.3) vs. Ctrl 10.0% (6.7; 13.3), data not shown) on thrombocytes were largely unaltered. PAF elicited a moderate increase in MMP9 and a marked increase in NE, but neither in IL‐6 nor in IL‐8 (Figure 4).

Exposure to PAF and, to a greater extent, LPS, significantly increased CD11b expression on granulocytes (Figure 5). A similar pattern was observed for other neutrophil activation markers; for example, a pronounced increase in CD10 expression or a strong decrease in CD62L (Figure S12). However, PAF but not LPS induced PNC formation. PAF and LPS increased both phagocytic activity and ROS generation. In most scenarios, additional stimulation with fMLF but not PAF in vitro displayed additive effects. Notably, previous exposure to LPS ex vivo did not prevent PNC formation by additional stimulation with PAF in vitro.

## 4 Discussion

PAF, as an inflammatory mediator, activates various cells, including neutrophils. This is in accordance with previous studies that demonstrated PAF‐induced upregulation of the integrin receptor CD11b and shedding of the selectin receptor CD62L [10, 44]. The PAF‐induced changes in phenotype were correlated in previous studies with increased neutrophil migratory activity (CD11b, CD62L, CD10) [44, 45, 46], an increase in IL‐8 secretion by crosslinking of CD66b, and FcγRIII upregulation (CD16) [47]. This change in phenotype coincided with increased phagocytic activity and ROS generation in vitro and ex vivo. These aspects, together with time‐ and dose‐related aspects, were generated to confirm data previously reported by others as discussed above. To the knowledge of the authors, the following aspects contain novel elements not previously demonstrated in the literature: (a) comparison of the PAF‐induced change in neutrophils in comparison to a broad plethora of other inflammatory mediators, (b) the in‐depth analysis of the impact of PNC formation of neutrophils as induced by PAF (comparison of PMN and PNC), (c) the impact of several pharmacological modulators on PAF‐induced neutrophil activity and PNC formation and (d) the transfer in a clinically relevant, well‐described [10, 29, 48] animal‐free ex vivo whole blood model of PAF‐induced acute inflammation.

The present manuscript compared PAF with other proinflammatory mediators like chemokines activating neutrophils via binding to specific G‐protein coupled receptors, including the chemotactic lipid LTB_4_ known as an intermediate target chemokine and end‐target formyl peptides including fMLF [28], as well as the cytokine TNF known as quite persuasive agent for neutrophil function [49]. PAF exhibited a unique profile of changing both neutrophil phenotype and activity while in parallel triggering PNC formation. In this context, the comparison to mediators more in association with platelet activation like the potent platelet agonist thrombin that leads to platelet activation and the release of ADP from the platelet dense granules along with TXA2, an eicosanoid synthesised by the through platelets activated arachidonic acid metabolism [50], emphasise even more the unique and important role of PAF. Adrenaline was included because its release often coincides with neutrophil (e.g., fever) and/or platelet activation (e.g., haemorrhage), however, in accordance to previous results by others [51], did not evoke changes in neutrophils and/or platelets as analysed in the present paper. As a limitation, it should be noted that the comparison of the mediators has a rather exploratory character (number of read‐out parameters, sample size).

PAF‐induced changes displayed clear dose‐ and time‐dependence. Neutrophil activation occurred in the low‐medium nanomolar ranges. The systemic determination of the PAF concentration is difficult due to its high turnover, local differences in concentrations and the property of PAF (with various related substances) as a lipid mediator [1]. However, these concentrations have also been reported in healthy individuals, and significant increased levels were observed in patients with sepsis and renal failure or allergic reactions [4, 52]. In addition, these changes in neutrophil phenotype and cell functions show similar timelines as our previous findings with regard to neutrophil cell physiology [10].

Moreover, PAF‐induced PNC formation has been previously postulated by us and others [10, 47]. PNC formation appears to include on a molecular level the interaction of several receptors and ligands, including PSGL‐1 with CD62P (P‐selectin), CD40 with CD40L and TREM1 with TREM1L, among others [33]. In this context, it is of note that neutrophils are able to produce PAF upon stimulation with fMLF or LTB_4_ [53], which might partly explain the (weak) stimulation of PNC formation by those mediators (Figure 1). Furthermore, one of the most important and prominent ones that the PNC formation depends on is CD62P on platelets with PSGL‐1 on neutrophils [33, 43] and the expression of CD62P is enhanced after PAF (Figure S11), ADP, or TXA_2_ stimulation by activating platelets but not by the chemokine fMLF. This further emphasises the important role of platelet activation and thereby the role of PAF in thromboinflammation. The interaction of (activated) platelets and neutrophils has been described to increase ROS generation and phagocytic activity [54, 55, 56]. The present study now confirms this for PAF stimulation. Of note, the increased phagocytic activity was sensitive toward targeting of PNC formation by iloprost or by blocking CD62P. Interestingly, ROS generation was not sensitive to the blocking of PNC formation, which is in contrast to a previous study reporting increased PNC‐dependent ROS generation upon stimulation with ADP [56]. The therapeutic relevance of iloprost as a platelet‐inhibiting reagent was also studied earlier in the onset of a systemic inflammation within a septic shock‐induced endotheliopathy [57] and COVID19‐related vasculopathy [58]. Our research emphasises in detail the relevance of iloprost on the proinflammatory mediator PAF that is known to have a non‐negligible relevance in those systemic inflammatory conditions. It is also of note that we were able to show the effects of iloprost on neutrophil function, for example, the phagocytic activity, in a micromolar range, which is comparable with regard to the applied concentrations in the studies that were discussed above. By demonstrating the ability of iloprost to prevent PAF‐induced PNC formation, the present study provides an interesting additional aspect of the application opportunities of this prostacyclin analog, which is by no means limited to the pure antithrombotic effect known for prostaglandin derivatives and may be extended relevantly to PAF‐driven acute inflammation.

It could be proposed that the inflammatory mediator involved in platelet and/or neutrophil stimulation may be the relevant component regarding the mode of platelet–neutrophil interaction (direct, humoral, via extracellular vesicles, etc.). However, the applied experimental conditions on naturally unstable cells such as neutrophils and platelets may in particular also play an important role when comparing different studies. The formation of PNCs using PAF led to pronounced neutrophil function (including ROS generation and phagocytosis) but not to an enhanced expression of activation markers (including CD11b). The increased generation of ROS might be a relevant link to pathophysiologies coinciding with an increased PAF generation [59]. However, the PNC‐related increased activities of ROS generation and phagocytic activity were not largely affected by blocking the formation of PNCs by iloprost or blocking CD62P. Therefore, further research needs to elucidate whether direct cell–cell interactions and/or humoral effects (e.g., humoral mediators, extracellular vesicles) are responsible for PAF‐induced increases in neutrophil activity. These findings might be of special clinical interest, because iloprost is already in several countries in clinical use for other indications than modulating PNCs. Regarding CD11b, the results are in line with another study using fMLF to induce PNC formation [56]. In [56], the authors have also demonstrated, that the mediator ADP, in contrast to our findings regarding PAF, induces enhanced expression of CD11b in PNCs. This significant difference might be explained by the fact that ADP is mainly a platelet activating factor [50] and subsequently leads to PNC formation thereby causing increased CD11b expression by neutrophils in the complex. We have demonstrated a more pronounced activation of neutrophils not in complex with platelets by PAF or fMLF (illustrated by CD11b) in contrast to ADP (Figure 1). This suggests that a strong preceding activation of neutrophils by PAF or fMLF that is already known [10] limits further increased CD11b expression during PNC formation and might explain the less pronounced CD11b expression. Based on the current findings and to the knowledge of the authors, one potential explanation is that either parallel actions of mediators (e.g., neutrophils, which have been primed, interacting with platelets which bind to neutrophils, both due to the same mediator) or singular actions of mediators (e.g., a mediator only activating platelets but not neutrophils, causing activated platelets interacting with neutrophils) might explain the differences in the activation pattern elicited by PAF, ADP and other inflammatory mediators.

The present study has several strengths and limitations. While the study adds multiple novel aspects to PAF‐driven neutrophil activation, also in the context of PNC formation, and offers specific pharmaceutical targeting options, the analyses performed included samples from only healthy and young subjects. Therefore, follow‐up studies need to confirm the findings in blood from older individuals with and without comorbidities. Also, for example in the animal‐free ex vivo whole blood model, further time aspects should be investigated (longer/shorter exposition of PAF to whole blood), further exploration of different PAF concentrations, interactions of PAF with corresponding receptors, and the combination of inflammatory mediators. The majority of the experiments were conducted in vitro, focusing on flow cytometry as the main method. Even so, the key findings were successfully reproduced in a clinically relevant ex vivo whole blood model of PAF/LPS‐induced acute inflammation. The application of the whole blood model enabled findings in an environment of prolonged PAF exposure without systemic anticoagulation. For example, the present findings of PAF‐induced increased NE production link PAF to NE with the properties of PAF as a key driver of tissue injury and systemic inflammation [60]. Changes in neutrophil phenotype regarding PNC formation were confirmed using two different panels of antibodies and d. Further studies should confirm the findings with another method, such as imaging cytometry or fluorescence microscopy, and validate the results in patients with acute and/or chronic inflammation.

## 5 Conclusion

PAF triggers a myriad of neutrophil actions, including changes in the phenotype, increased cellular activity and PNC formation. This study links PNCs to increased neutrophil activity and provides novel insights into potential therapeutic approaches to modulate excessive inflammation, for example, during sepsis. The present findings should be confirmed in blood from patients with systemic inflammation, for example, after severe trauma or during sepsis, and might then provide novel pharmacological approaches to modulate acute thromboinflammation.

## Author Contributions

Conceptualization: Lisa Wohlgemuth, Markus Huber‐Lang, and David Alexander Christian Messerer. Data curation: Lisa Wohlgemuth, Christiane Leonie Knapp, Laura Vidoni, and David Alexander Christian Messerer. Formal analysis: Lisa Wohlgemuth, Christiane Leonie Knapp, Laura Vidoni, Paul Müller, and David Alexander Christian Messerer. Funding acquisition: Markus Huber‐Lang and David Alexander Christian Messerer. Investigation: Lisa Wohlgemuth, Christiane Leonie Knapp, Laura Vidoni, Adam Omar Khalaf Mohamed, and Annika Dietz. Methodology: Lisa Wohlgemuth, Christiane Leonie Knapp, Stefan Hug, Alexander Elias Paul Stratmann, Laura Stukan, Larissa Melina Höpfer, Frederik Münnich, Bertram Dietrich Thomaß, Michael Ruhland, Markus Huber‐Lang, and David Alexander Christian Messerer. Project administration: David Alexander Christian Messerer. Resources: Markus Huber‐Lang and David Alexander Christian Messerer. Visualisation: Lisa Wohlgemuth, Christiane Leonie Knapp, Laura Vidoni, Paul Müller, Annika Dietz, Michael Ruhland, and David Alexander Christian Messerer. Writing – original draft: Lisa Wohlgemuth and David Alexander Christian Messerer. Writing – review and editing: All authors.

## Conflicts of Interest

The authors declare that the research was conducted in the absence of any commercial or financial relationships that could be construed as a potential conflicts of interest. A subset of the presented data has been generated within a thesis of C.L.K. and has previously been made publicly available https://doi.org/10.18725/OPARU‐52547.

## Supporting information


Data S1:

## Data Availability

The data that support the findings of this study are available from the corresponding author upon reasonable request.
