# Supplementary material for: Platelet‐Activating Factor Promotes Neutrophil Activation and Platelet–Neutrophil Complex Formation
Source: Scand J Immunol. 2025 Jul 28;102(2):e70044. doi: 10.1111/sji.70044 (PMC12304291; doi:10.1111/sji.70044)
Supplement: Supplementary file 1 — Data S1: [file SJI-102-e70044-s001.docx]

# Supplemental Material

|  | **0**^–^ | **Sham** | **PAF** | **LPS** |
| --- | --- | --- | --- | --- |
| **pO_2_ [mmHg]** | 37.1 (27.0; 42.7) | 62.4 (43.7; 77.1) | 57.8 (40.4; 79.0) | 73.1 (49.8; 95.2) |
| **pCO_2_ [mmHg]** | 42.0 (40.7; 47.3) | 40.6 (38.2; 43.3) | 41.2 (39.8; 44.2) | 39.7 (35.3; 45.2) |
| **pH-value** | 7.37 (7.33; 7.39) | 7.36 (7.32; 7.38) | 7.35 (7.32; 7.38) | 7.36 (7.32; 7.39) |
| **Potassium [mmol/L]** | 3.7 (3.4; 3.9) | 3.4 (3.2; 3.8) | 3.4 (3.2; 3.6) | 3.4 (3.3; 3.8) |
| **Sodium [mmol/L]** | 148.0 (147.0; 150.0) | 149.0 (148.5; 150.5) | 149.0 (147.0; 150.0) | 149.0 (147.5; 153.5) |
| **Glucose [mg/dL]** | 86.0 (81.3; 88.3) | 76.5 (72.0; 79.3) | 73.5 (65.0; 75.3) | 73.0 (68.5; 76.0) |
| **Lactate [mmol/L]** | 1.0 (0.9; 1.1) | 2.1 (2.0; 2.3) | 2.4 (2.3; 2.5) | 2.5 (2.2; 2.7) |
| **Thrombocytes [G/L]** | 172.0 (131.5; 216.5) | 169.0 (135.5; 196.5) | 170.0 (137.5; 220.0) | 160.0 (121.0; 186.5) |
| **Erythrocytes [T/L]** | 4.2 (3.9; 4.4) | 4.1 (4.0; 4.5) | 4.1 (4.0; 4.4) | 4.1 (3.9; 4.4) |
| **Hemoglobin [g/dL]** | 12.2 (11.2; 13.2) | 12.2 (11.4; 13.3) | 12.3 (11.2; 13.3) | 12.1 (11.1; 13.2) |
| **Hematocrit [%]** | 46.7 (43.0; 49.6) | 48.2 (42.0; 49.4) | 47.7 (41.4; 50.2) | 47.1 (43.2; 50.8) |
| **Leukocytes [G/L]** | 5.0 (4.3; 5.9) | 4.8 (4.3; 5.9) | 5.1 (4.5; 6.5) | 4.8 (4.2; 5.6) |
| **Basophiles [%]** | 0.5 (0.25; 0.9) | 0.6 (0.4; 0.7) | 0.5 (0.4; 0.8) | 0.4 (0.2; 0.9) |
| **Eosinophiles [%]** | 3.0 (0.9; 3.8) | 3.2 (1.0; 4.0) | 2.5 (1.1; 3.5) | 2.9 (1.0; 3.8) |
| **Neutrophiles [%]** | 53.0 (49.9; 57.7) | 52.5 (49.8; 57.7) | 53.3 (49.2; 60.6) | 53.6 (51.9; 59.8) |
| **Monocytes [%]** | 8.4 (7.3; 10.1) | 8.8 (7.7; 10.1) | 8.6 (7.7; 10.2) | 8.5 (8.0; 10.3) |
| **Lymphocytes [%]** | 33.1 (30.9; 37.0) | 32.6 (31.6; 38.2) | 32.4 (29.9; 38.3) | 31.0 (29.0; 35.9) |

**Supplemental Table 1:** Changes in electrolyte concentrations, metabolism, and cell count before and after exposure of whole blood to the ex vivo circuit system. All parameters like oxygen partial pressure (pO_2_) and carbon dioxide partial pressure (pCO_2_) were collected before exposure to the ex vivo whole blood system (0^–^) and after a 1-hour incubation with PBS^++^ as Ctrl (Sham), PAF, or LPS. The following concentrations were used: PAF 1 µM, LPS 100 ng/mL. n = 9 − 10, median ± interquartile range.

|  | **Antigen** | **Fluorochrome** | **Final concentration** | **Cat number**  **Antibody**  **Isotype** | **Vendor** |
| --- | --- | --- | --- | --- | --- |
| **“ref”** |  |  |  |  |  |
| phenotype | CD10 (Neprilysin) | PE-Cy7 | 120 ng/mL | #312214  #400118 | BioLegend |
|  | CD11b (Integrin αM) | APC | 600 ng/mL | #101212  #400612 | BioLegend |
|  | CD15 (SSEA-1) | FITC | 50 ng/mL | #301904  #401606 | BioLegend |
|  | CD16 (FcγRIII) | PerCP | 200 ng/mL | #302030  #400148 | BioLegend |
|  | CD62L (L-selectin) | PE | 10 ng/mL | #304806  #400112 | BioLegend |
|  | CD66b (CEACAM8) | APC-Cy7 | 1 µg/mL | #305126  No isotype | BioLegend |
| function | Phagocytosis beads (Phagocytic activity) | BV421 | 100 µL/mL | #18339-10 | Polysciences Inc. |
|  | CellROX Deep Red (ROS generation) | APC | 5 µM | #C10422 | Thermo Fisher |
|  | CD61 (GPIIIa) | PerCP | 2 µg/mL | #336410  #400148 | BioLegend |
| **“conf1”** |  |  |  |  |  |
|  | CD11b (Integrin αM) | FITC | 16 µg/mL | #301330  #400110 | BioLegend |
|  | Phagocytosis beads (Phagocytic activity) | BV421 | 100 µL/mL | #18339-10 | Polysciences Inc. |
|  | CellROX Deep Red (ROS generation) | APC | 25 µM | #C10422 | Thermo Fisher |
|  | CD61 (GPIIIa) | PerCP Cy5.5 | 275 ng/mL | #336418  #400150 | BioLegend |
| **“conf2”** |  |  |  |  |  |
|  | CD10 (Neprilysin) | PE-Cy7 | 10 µg/mL | #565282  #557872 | BD Biosciences |
|  | CD11b (Integrin αM) | BV711 | 37.8 µg/mL | #563168  #563045 | BD Biosciences |
|  | CD182 (CXCR2) | APC | 2.5 µg/mL | #551127  #555751 | BD Biosciences |
|  | CD35 (Complement receptor 1) | BV605 | 50 µg/mL | #744276  #562652 | BD Biosciences |
|  | CD62L (L-selectin) | BV421 | 3.4 µg/mL | #563862  #562438 | BD Biosciences |
|  | CD66b (CEACAM8) | PerCP-Cy5.5 | 2.5 µg/mL | #562254  #560857 | BD Biosciences |
|  | CD61 (GPIIIa) | FITC | 2.4 µg/mL | #336404  #400110 | BioLegend |

**Supplemental Table 2**: Summary of fluorescent probes used for measurement of the PAF-induced changes in vitro. Isotype controls were used in the same final concentration as the corresponding antibody. Vendors included are BioLegend (San Diego, CA, USA), BD Biosciences, San Jose, USA, Thermo Fisher Scientific, Darmstadt, Germany and Polysciences Inc., Warrington, PA, USA. Platelet-Neutrophil Complexes (PNCs) were identified by CD61 and a representative histogram with the corresponding gating strategy is shown in Figure 4C.

**
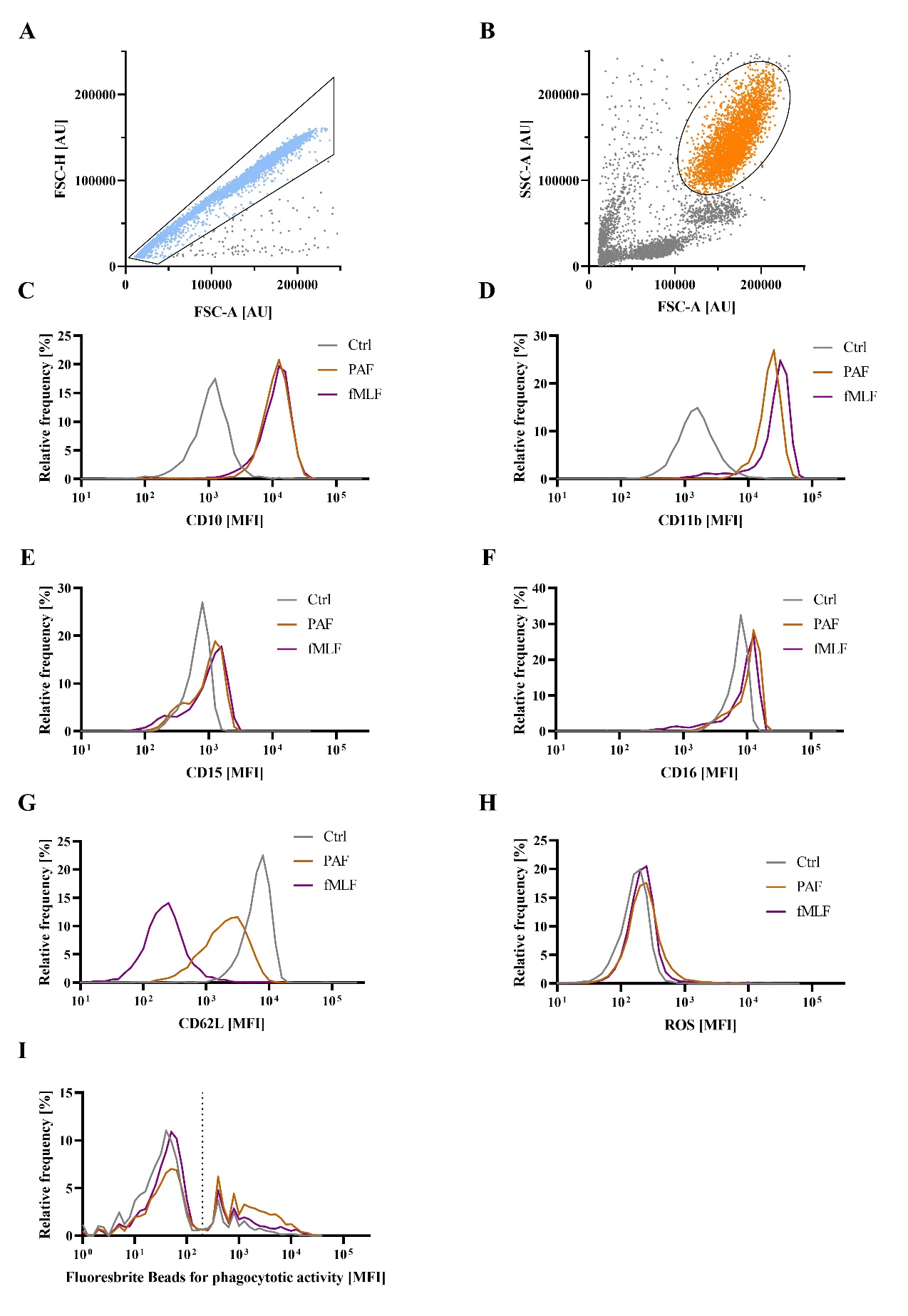
 Supplemental Figure 1:** Representative gating strategy and histograms.


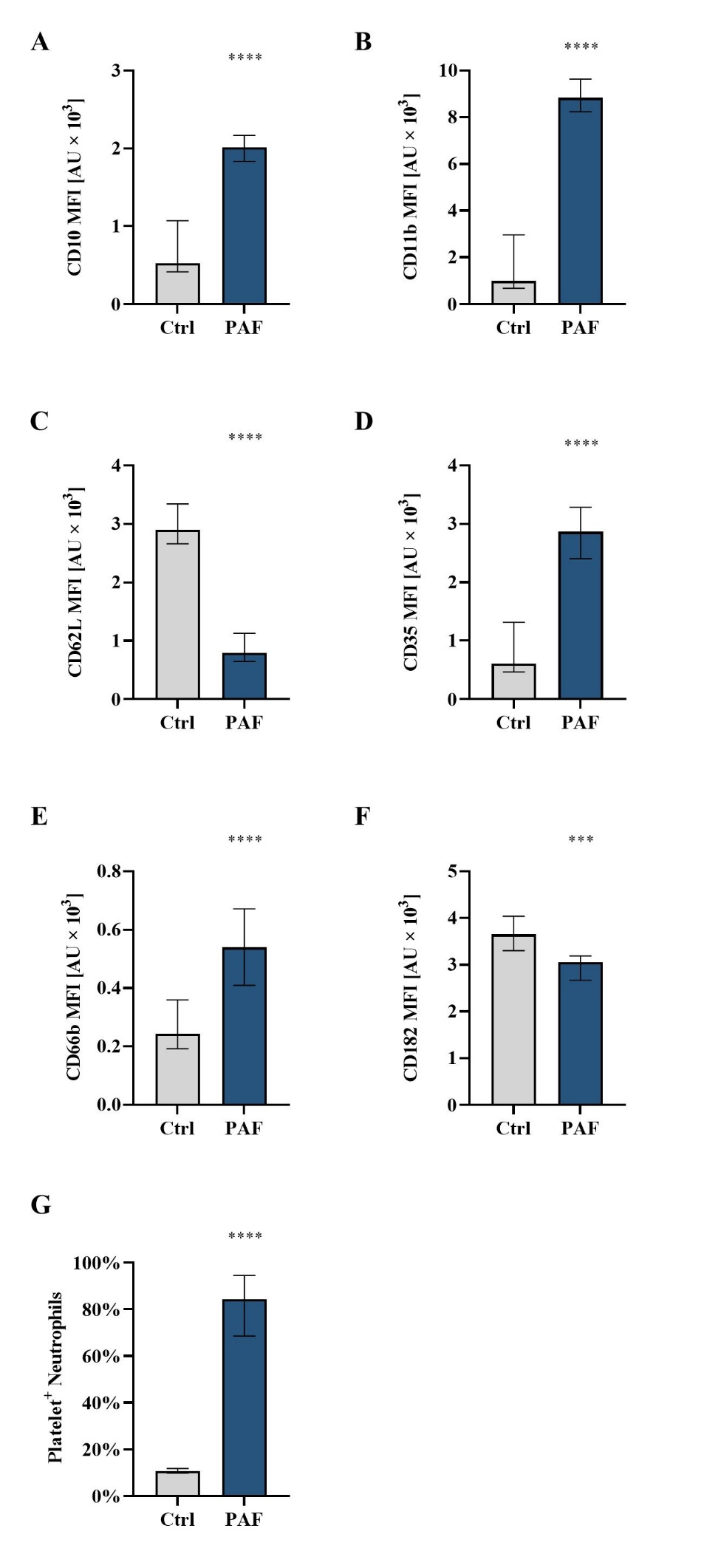


**Supplemental Figure 2:** PAF-induced activation of neutrophils in diluted whole blood in vitro within the conf2 data set. **A**) CD10, **B**) CD11b, **C**) CD62L, **D**) CD35, **E**) CD66b, **F**) CD182, and **G**) platelet-neutrophil complex (PNC) formation. **A**–**F**) Y-axis reports median fluorescence intensity (MFI) for all CD molecules, **G**) shows the percent positive neutrophils for PNC. The used PAF concentration was 1 µM. n = 10, median ± interquartile range. ***, **** = p < 0.001, < 0.0001, respectively. Mann-Whitney-U-test for neutrophils stimulated with PBS^++^ as control (Ctrl) vs. stimulated with PAF.
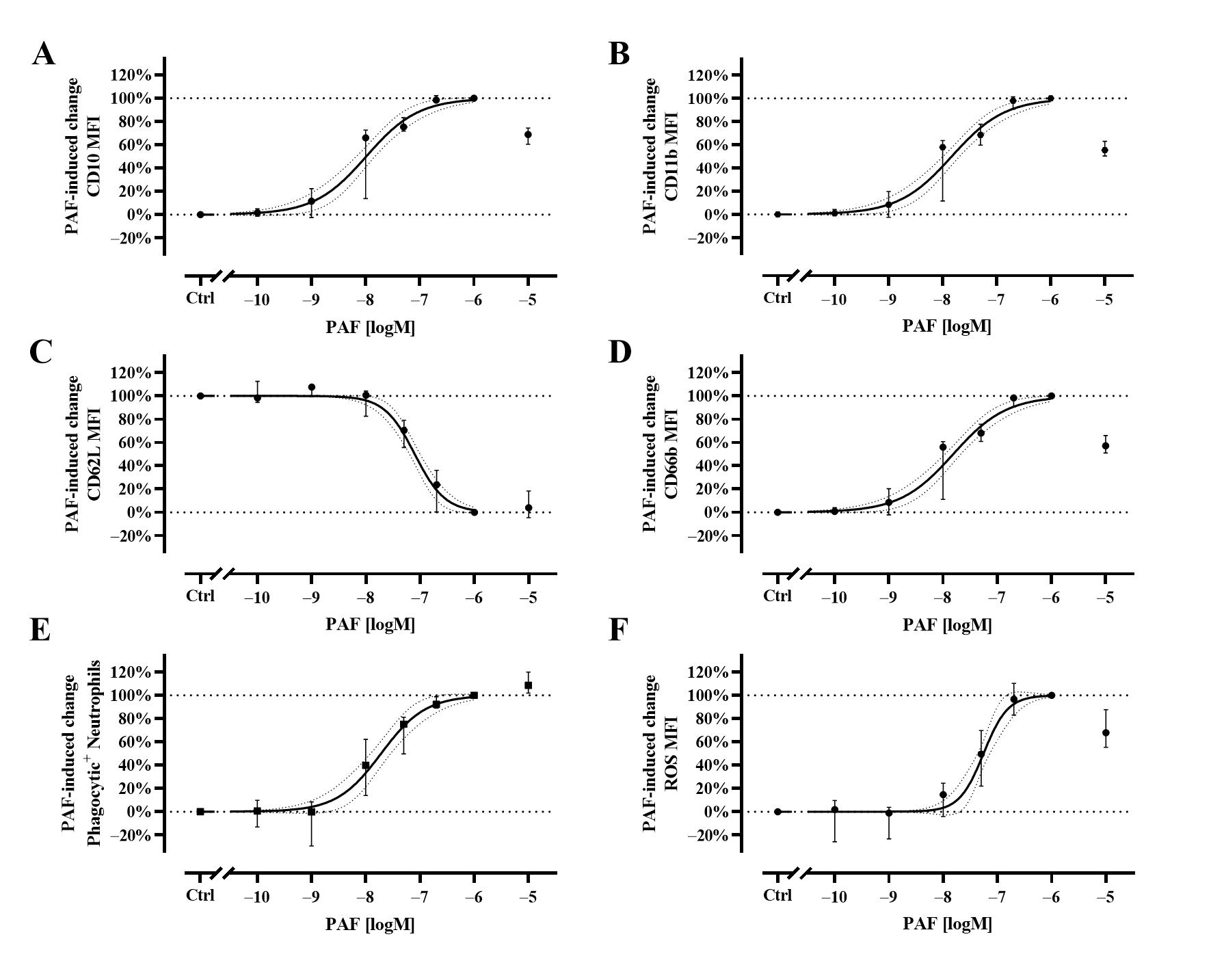


**Supplemental Figure 3:** Concentration-dependency of PAF-induced changes of neutrophil activity in diluted whole blood in vitro. **A**) CD10, **B**) CD11b, **C**) CD62L, **D**) CD66b, **E**) phagocytic activity, and **F**) generation of reactive oxygen species (ROS). **A**–**D**), **F**) Y-axis reports normalized median fluorescence intensity (MFI) dose-dependency changes for all CD molecules and ROS production; in **E**) the normalized phagocytic activity is displayed. Neutrophils were stimulated with PAF concentrations from 10 µM to 100 pM. The value 0% corresponds to neutrophils stimulated with PBS^++^ as control (Ctrl) and 100% to the maximum effect of neutrophils stimulated with PAF. n = 5 − 6, median ± interquartile range.

**
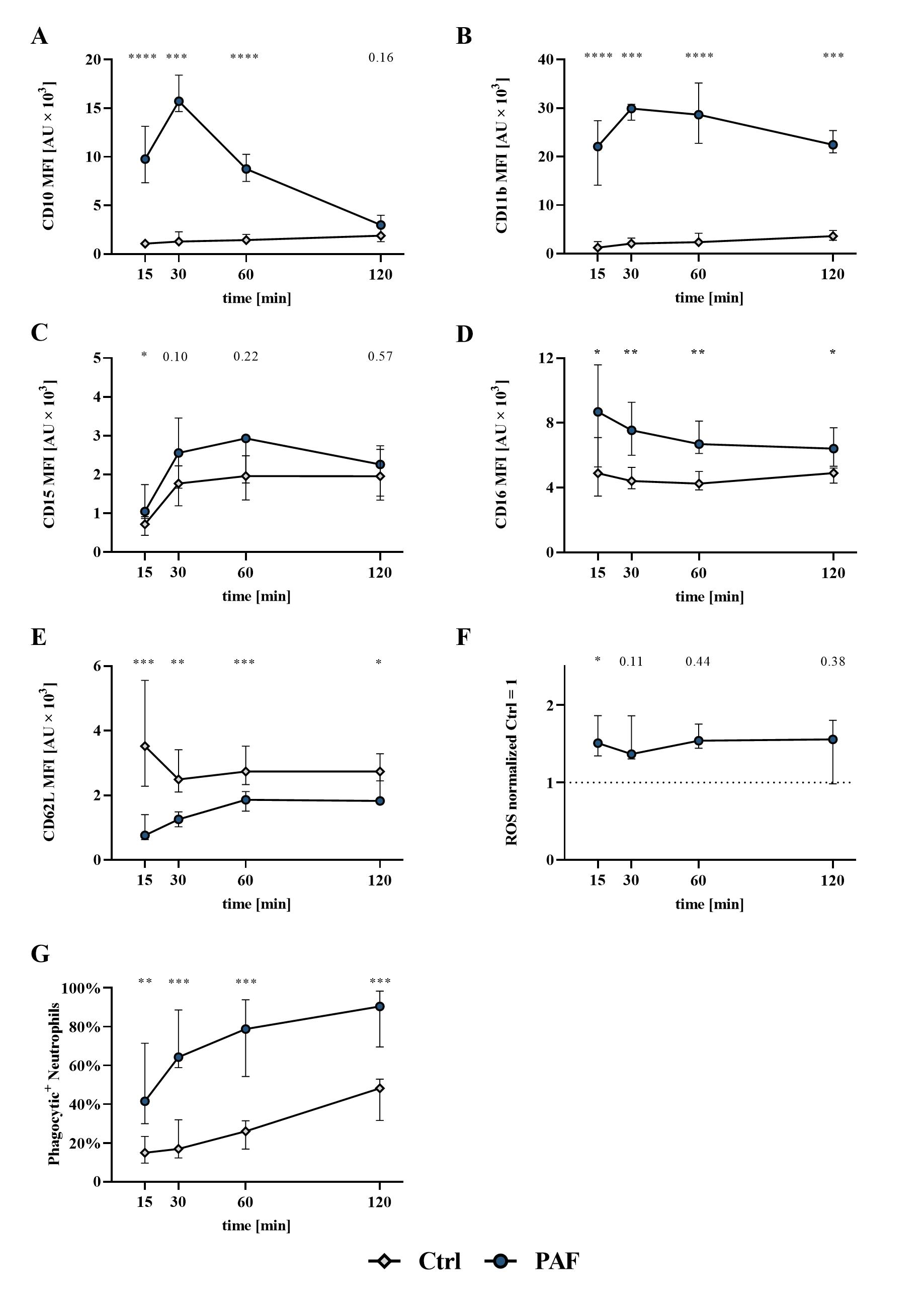
 Supplemental Figure 4:** Time-dependency of the neutrophil response in diluted whole blood after exposure to PAF in vitro. **A**) CD10, **B**) CD11b, **C**) CD15, **D**) CD16, **E**) CD62L, **F**) generation of reactive oxygen species (ROS), and **G**) phagocytic activity. **A**–**E**) Y-axis reports median fluorescence intensity (MFI) for all CD molecules, **F**) shows the increase in ROS production normalized to unstimulated neutrophils (Ctrl = 1.0), and **G**) shows the percent positive neutrophils for phagocytosis. The used PAF concentration was 1 µM. n = 7 − 9, median ± interquartile range. *, **, ***, **** = p < 0.05, < 0.01, < 0.001, < 0.0001, respectively. Mann-Whitney-U-test in **A–E**, **G** and Wilcoxon test in **F** for neutrophils stimulated with PBS^++^ as control (Ctrl) vs. PAF for each time point (15 min, 30 min, 60 min, and 120 min).


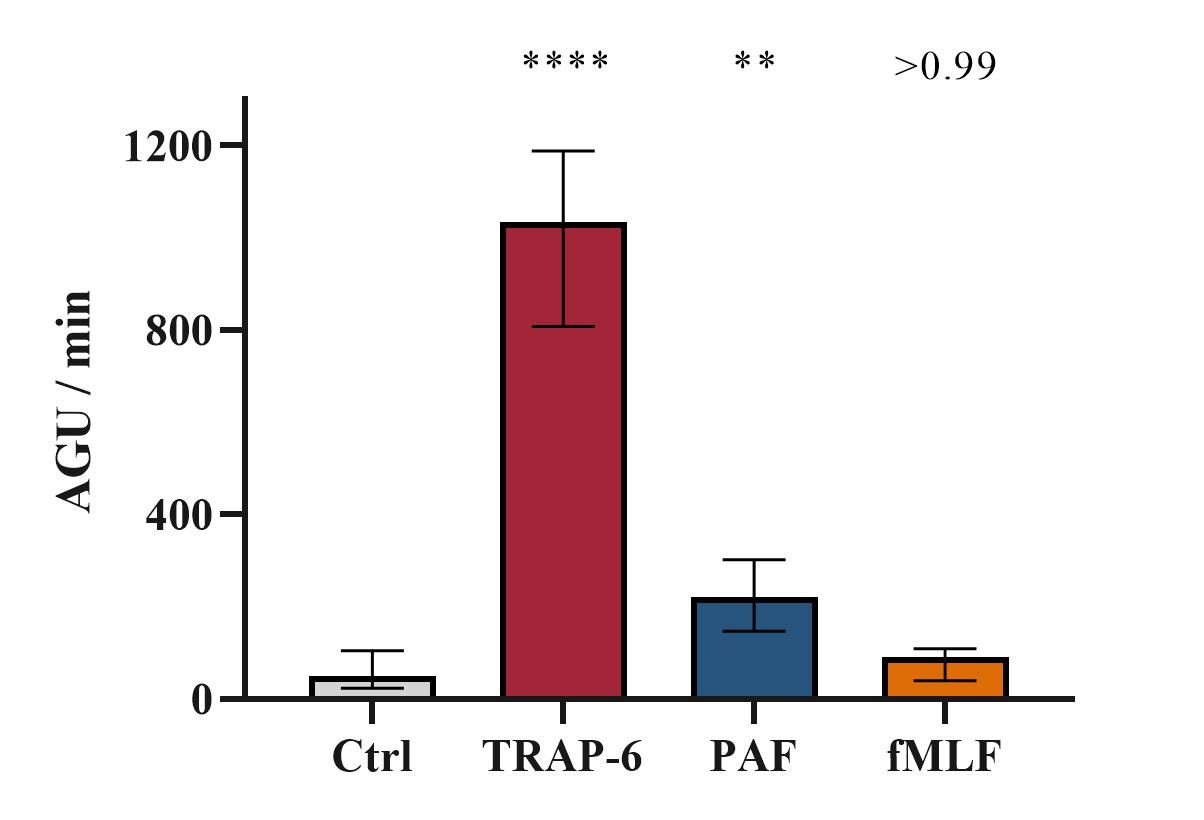


**Supplemental Figure 5:** PAF-induced effects on platelet aggregation in diluted whole blood in vitro measured by impedance aggregometry. Whole blood was either stimulated with PBS^++^ as Ctrl, TRAP-6, PAF, or fMLF. Y-axis reports aggregation units (AGU) per minute. The following concentrations were used: thrombin receptor-activating peptide 6 (TRAP-6) 15 µM, PAF 1 µM, and fMLF 1 µM. n = 13, median ± interquartile range. **, *** = p < 0.01, < 0.001, respectively. Kruskal-Wallis-test with uncorrected Dunn’s test for neutrophils incubated with PBS^++^ as control (Ctrl) vs. neutrophils incubated with TRAP-6, PAF, or fMLF.


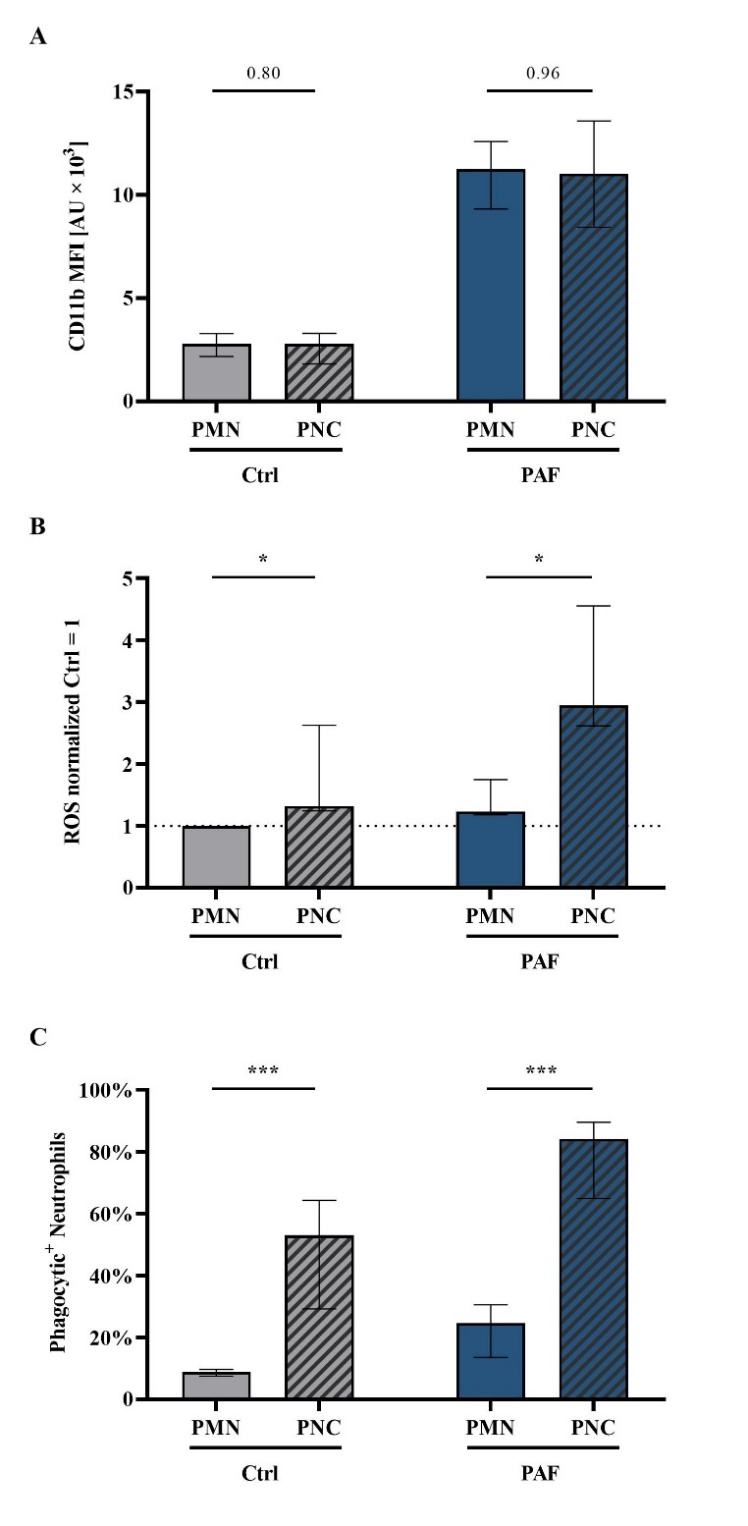


**Supplemental Figure 6:** Confirmation of platelet-neutrophil complex (PNC) formation affecting neutrophil function but not phenotype in diluted whole blood in vitro after PAF stimulation compared to neutrophils not in complex with platelets (PMNs) within conf1. **A**) CD11b, **B**) generation of reactive oxygen species (ROS), and **C**) phagocytic activity. **A**) Y-axis reports median fluorescence intensity (MFI), **B**) shows the increase in ROS production normalized to unstimulated PMN (Ctrl = 1.0), and **C**) shows the percent positive neutrophils for phagocytosis. The used PAF concentration was 1 µM. n = 8, median ± interquartile range. *, *** = p < 0.05, < 0.001, respectively. Mann-Whitney-U-test in **A**, **C** and Wilcoxon-test in **B**. PMN stimulated with PBS^++^ as control (Ctrl) vs. PNC stimulated with PBS^++^ (Ctrl) and PMN stimulated with PAF vs. PNC stimulated with PAF.
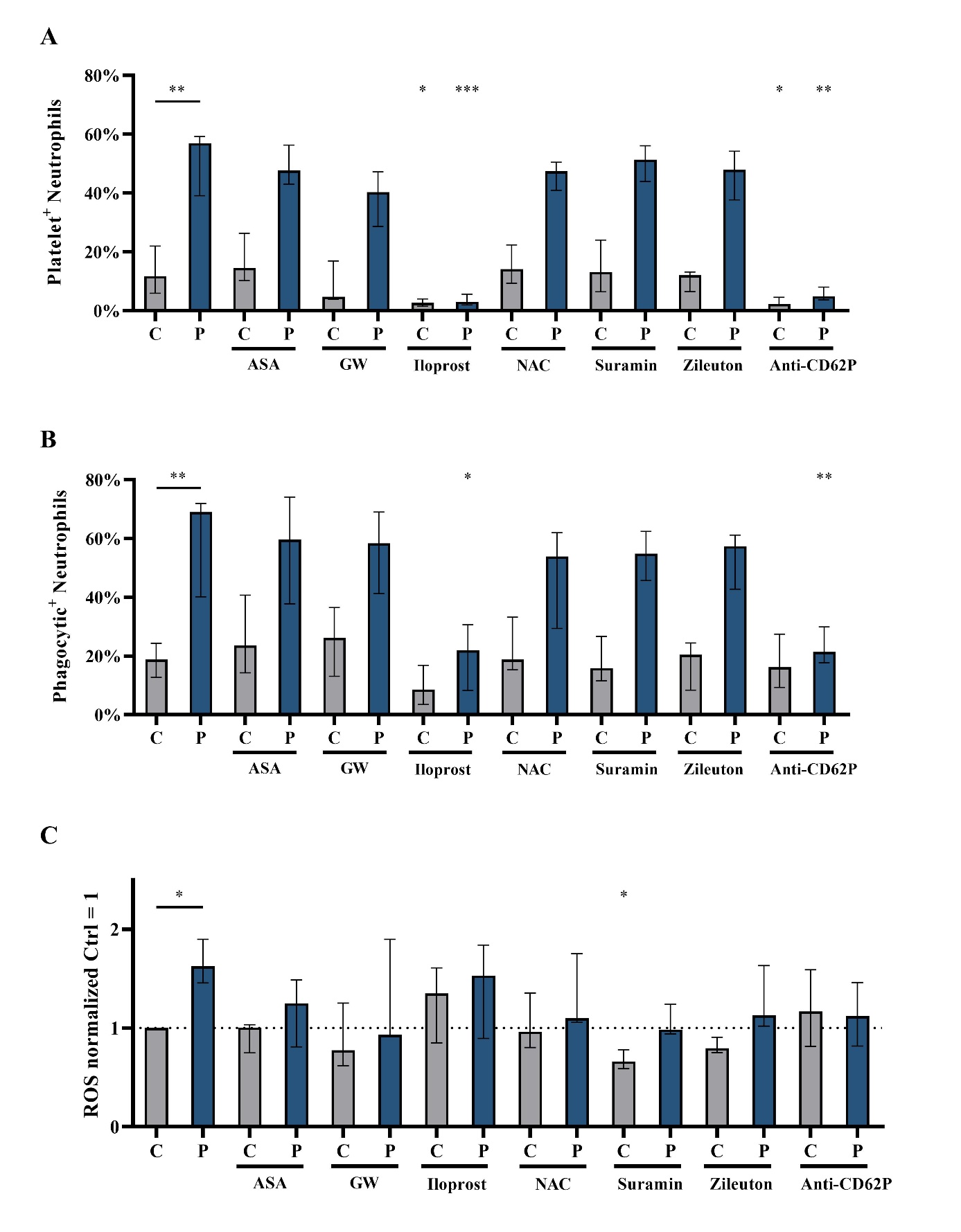
**Supplemental** **Figure 7:** Pharmacological characterization of the PAF-induced effects on neutrophil function in diluted whole blood in vitro. Impact of screened pharmacological agents on **A**) platelet-neutrophil complex (PNC) formation, **B**) phagocytic activity, and **C**) generation of reactive oxygen species (ROS). The following concentrations were used: PAF (P) 1 µM, acetylsalicylic acid (ASA) 1.7 µM, GW4869 (GW) 2 µM, N-acetyl-L-cysteine (NAC) 2 mM, suramin 100 µM, zileuton 2.5 µM, and anti-CD62P antibody (Anti-CD62P) 60 nM. n = 5 − 7, median ± interquartile range. *, **, *** = p < 0.05, < 0.01, < 0.001, respectively, if not indicated otherwise, versus control neutrophils without exposure to a pharmacological modulator (first column of each graph). Percentage of positive neutrophils for PNC formation in **A**) and for phagocytic activity in **B**). **C**) Shows the increase in ROS production normalized to unstimulated neutrophils (Ctrl = 1.0). Mann-Whitney-U-test **A** – **B** and Wilcoxon-test **C** applied to values before normalization for neutrophils stimulated with only PBS^++^ as control (Ctrl, C) vs. neutrophils stimulated with only PAF. Kruskal-Wallis-test **A** – **B** and Friedmann-test **C** for absolute values with uncorrected Dunn’s test of neutrophils stimulated with PBS^++^ (Ctrl) among themselves and the same for neutrophils stimulated with PAF.


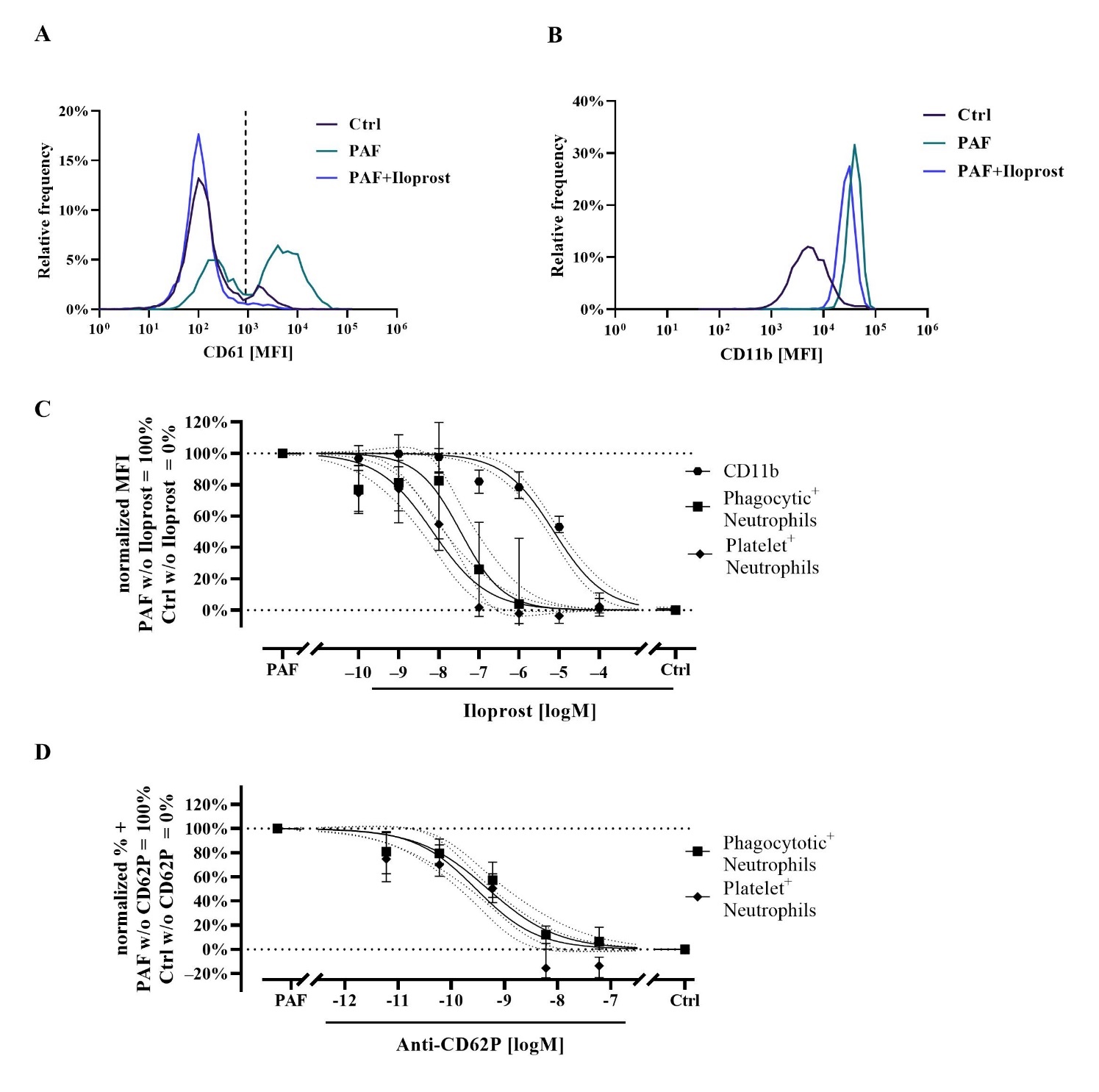
 **Supplemental Figure 8:** Concentration-dependency of iloprost-induced changes after PAF stimulation of neutrophil activity and function in diluted whole blood in vitro. **A**) Representative flow cytometry gating for platelet-neutrophil complexes (PNCs) using CD61 median fluorescence intensity (MFI) and **B**) for neutrophil activation using CD11b MFI after stimulation with PBS^++^ as control (Ctrl), PAF, or PAF and subsequently iloprost. **C**) Effects on neutrophil activity (CD11b) and neutrophil function (percentage of PNCs and phagocytic positive neutrophils) stimulated with PAF (1 µM) and subsequently with iloprost (100 µM – 100 pM). **D**) Effects on percentage of PNCs and phagocytic positive neutrophils stimulated with PAF (1 µM) and subsequently with CD62P antibody (60 nM – 6 pM). The value 0% corresponds to neutrophils stimulated with PBS^++^ as control (Ctrl) and 100% the maximum effect of neutrophils stimulated with PAF. n = 6 − 7, median ± interquartile range.


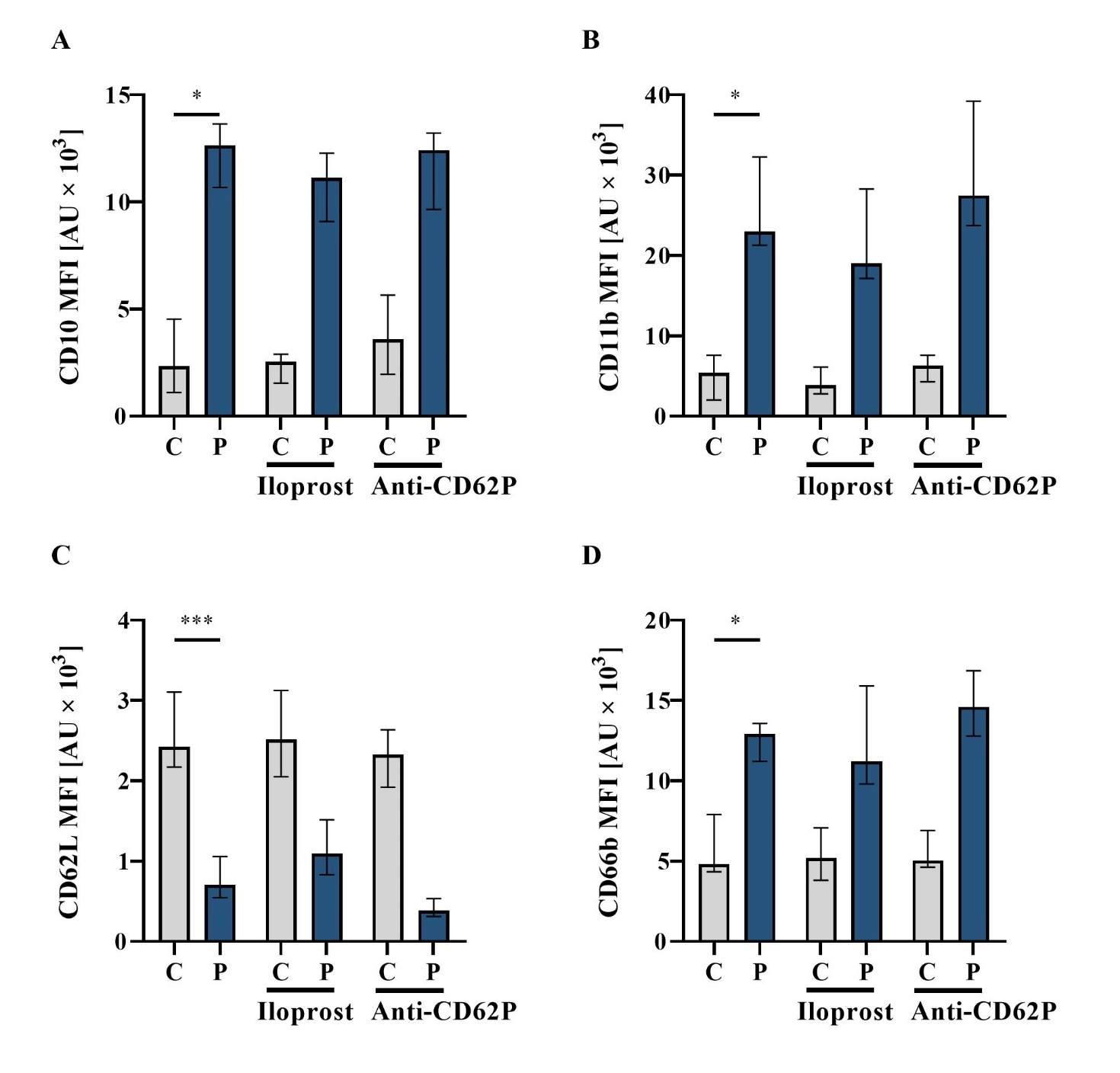


**Supplemental Figure 9:** Pharmacological agents iloprost and anti-CD62P do not modulate the PAF-induced effects on neutrophil phenotype in diluted whole blood in vitro. Impact of screened pharmacological agents on **A**) CD10, **B**) CD11b, **C**) CD62L, and **D**) CD66b. Y-axis reports the median fluorescence intensity (MFI) for all CD molecules. The used PAF concentration was 1 µM. n = 6 − 7, median ± interquartile range. *, ***, = p < 0.05, < 0.001, respectively. Kruskal-Wallis-test with uncorrected Dunn’s test for neutrophils incubated with PBS^++^ as control (Ctrl, C) vs. neutrophils incubated with PAF (P).


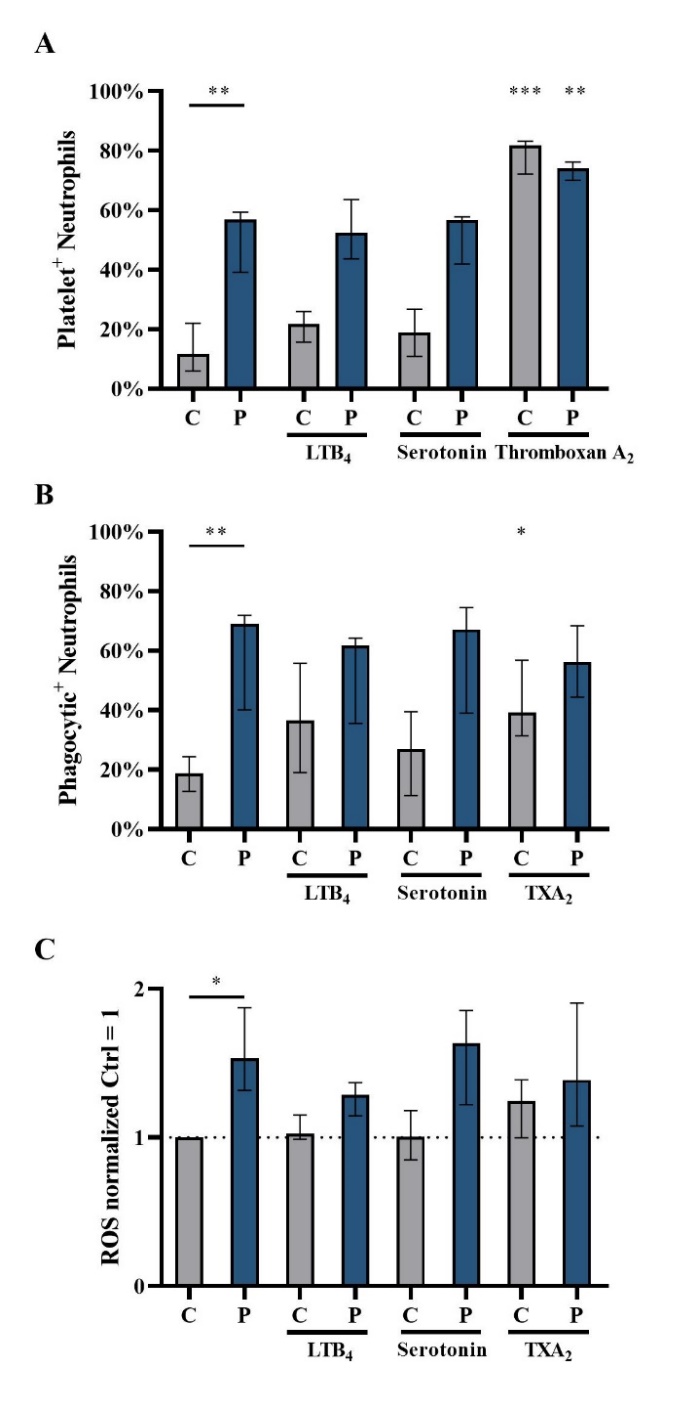


**Supplemental Figure 10:** Pharmacological characterization of the PAF-induced effects on neutrophil function in diluted whole blood in vitro. Impact of screened pharmacological agents on **A**) platelet-neutrophil complex (PNC) formation, **B**) phagocytic activity, and **C**) generation of reactive oxygen species (ROS). The following concentrations were used: PAF (P) 1 µM, leukotriene B_4_ (LTB_4_) 100 nM, serotonin 500 ng/mL, and thromboxane A_2_ (TXA_2_) 2 µM. n = 7, median ± interquartile range. *, **, *** = p < 0.05, < 0.01, < 0.001, respectively. Percent positive neutrophils for PNC formation in **A**) and phagocytic activity in **B**). **C**) Shows the increase in ROS production normalized to unstimulated PMN (Ctrl = 1.0). Mann-Whitney-U-test **A**, **B**) and Wilcoxon-test for absolute values **C**) for neutrophils stimulated with only PBS^++^ as control (Ctrl, C) vs. neutrophils stimulated with only PAF. Kruskal-Wallis-test **A**, **B** and Friedmann-test calculated for values before normalization in **C** with uncorrected Dunn’s test of neutrophils stimulated with PBS^++^ (Ctrl, C) among themselves and the same for neutrophils stimulated with PAF.


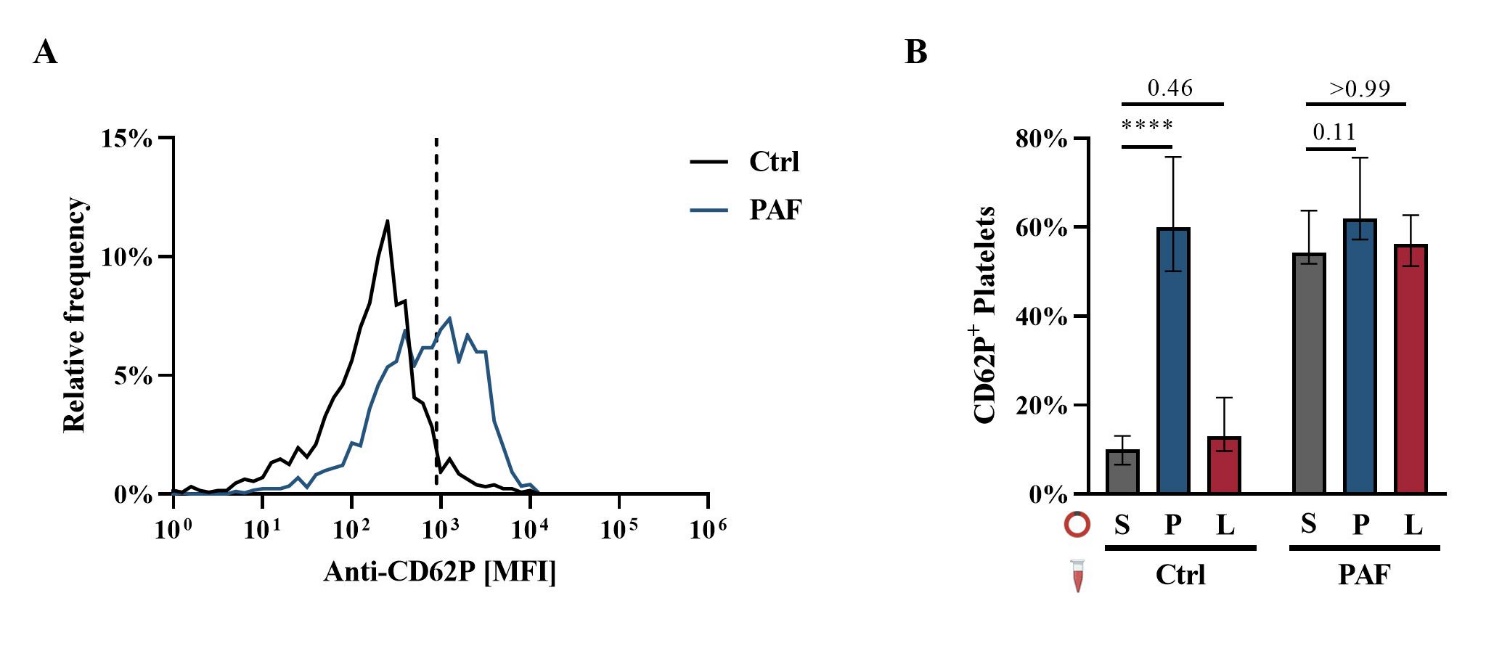


**Supplemental Figure 11:** PAF induces platelet activation within an animal-free ex vivo whole blood model. Human whole blood was either incubated within the ex vivo whole blood model for 1 h with PBS^++^ as Ctrl (Sham (S)), PAF (P), or LPS (L), or directly analyzed without exposure to the ex vivo whole blood model (0^−^) as indicated in Figure 7 A. Platelets in diluted whole blood were further stimulated with PBS^++^ as Ctrl or PAF in vitro for 15 min. **A**) Representative flow cytometry gating for platelets using CD62P median fluorescence intensity (MFI) after stimulation with PBS^++^ as control (Ctrl) or PAF. **B**) Summary data for the percentage of CD62P-positive platelets detected by flow cytometry. The following concentrations were used: PAF 1 µM, LPS 100 ng/mL. n = 8 − 9, median ± interquartile range. **** = p < 0.0001. Kruskal-Wallis-test with uncorrected Dunn’s test for neutrophils incubated with PBS^++^ as control (Ctrl) vs. neutrophils incubated with PAF or LPS.

**
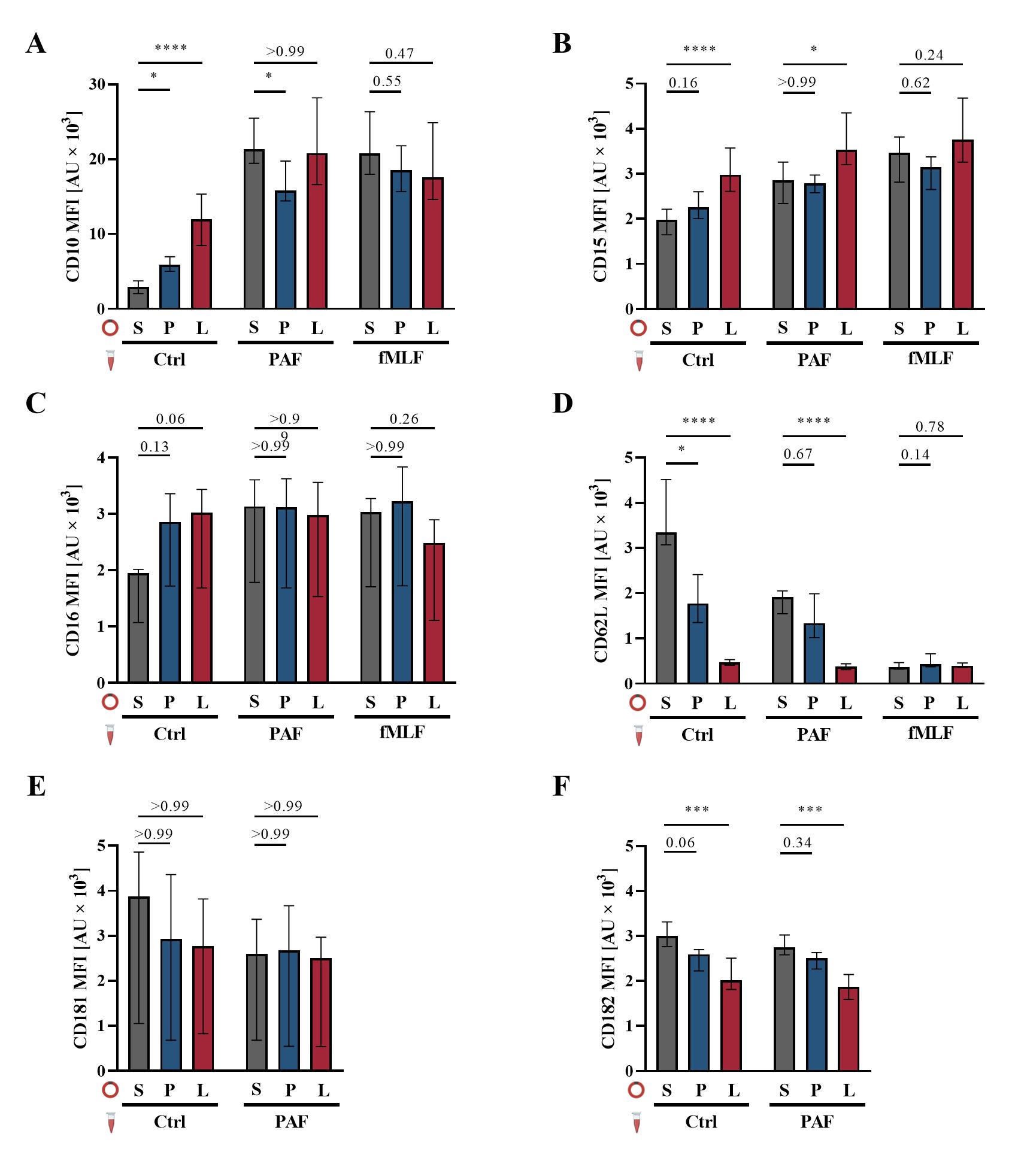
**

**Supplement Figure 12:** PAF-driven inflammation changes neutrophil phenotype within an animal-free ex vivo whole blood model. Human whole blood was either incubated within the ex vivo whole blood model for 1 h with PBS^++^ as Ctrl (Sham (S)), PAF (P), or LPS (L), or directly analyzed without exposure to the ex vivo whole blood model (0^−^) as indicated in Figure 7 A. Neutrophils in diluted whole blood were further stimulated with PBS^++^ as Ctrl, PAF, or fMLF in vitro for 15 min. **A**) CD10, **B**) CD15, **C**) CD16, **D**) CD62L, **E**) CD181, and **F**) CD182. Y-axis reports the median fluorescence intensity (MFI) for all CD molecules. The following concentrations were used: PAF 1 µM, fMLF 10 µM, and LPS 100 ng/mL. n = 10, median ± interquartile range. *, ***, **** = p < 0.05, < 0.001, < 0.0001, respectively. Kruskal-Wallis-test with uncorrected Dunn’s test for neutrophils incubated with PBS^++^ as control (Ctrl) vs. neutrophils incubated with PAF or LPS.
